# Household Transmission of SARS-CoV-2: A Systematic Review and Meta-analysis

**DOI:** 10.1001/jamanetworkopen.2020.31756

**Published:** 2020-12-14

**Authors:** Zachary J. Madewell, Yang Yang, Ira M. Longini, M. Elizabeth Halloran, Natalie E. Dean

**Affiliations:** 1 Department of Biostatistics, University of Florida, Gainesville; 2 Fred Hutchinson Cancer Research Center, Seattle, Washington; 3 Department of Biostatistics, University of Washington, Seattle

## Abstract

**Importance:**

Crowded indoor environments, such as households, are high-risk settings for the transmission of severe acute respiratory syndrome coronavirus 2 (SARS-CoV-2).

**Objectives:**

To examine evidence for household transmission of SARS-CoV-2, disaggregated by several covariates, and to compare it with other coronaviruses.

**Data Source:**

PubMed, searched through October 19, 2020. Search terms included *SARS-CoV-2* or *COVID-19* with *secondary attack rate*, *household*, *close contacts*, *contact transmission*, *contact attack rate*, or *family transmission*.

**Study Selection:**

All articles with original data for estimating household secondary attack rate were included. Case reports focusing on individual households and studies of close contacts that did not report secondary attack rates for household members were excluded.

**Data Extraction and Synthesis:**

Meta-analyses were done using a restricted maximum-likelihood estimator model to yield a point estimate and 95% CI for secondary attack rate for each subgroup analyzed, with a random effect for each study. To make comparisons across exposure types, study was treated as a random effect, and exposure type was a fixed moderator. The Preferred Reporting Items for Systematic Reviews and Meta-analyses (PRISMA) reporting guideline was followed.

**Main Outcomes and Measures:**

Secondary attack rate for SARS-CoV-2, disaggregated by covariates (ie, household or family contact, index case symptom status, adult or child contacts, contact sex, relationship to index case, adult or child index cases, index case sex, number of contacts in household) and for other coronaviruses.

**Results:**

A total of 54 relevant studies with 77 758 participants reporting household secondary transmission were identified. Estimated household secondary attack rate was 16.6% (95% CI, 14.0%-19.3%), higher than secondary attack rates for SARS-CoV (7.5%; 95% CI, 4.8%-10.7%) and MERS-CoV (4.7%; 95% CI, 0.9%-10.7%). Household secondary attack rates were increased from symptomatic index cases (18.0%; 95% CI, 14.2%-22.1%) than from asymptomatic index cases (0.7%; 95% CI, 0%-4.9%), to adult contacts (28.3%; 95% CI, 20.2%-37.1%) than to child contacts (16.8%; 95% CI, 12.3%-21.7%), to spouses (37.8%; 95% CI, 25.8%-50.5%) than to other family contacts (17.8%; 95% CI, 11.7%-24.8%), and in households with 1 contact (41.5%; 95% CI, 31.7%-51.7%) than in households with 3 or more contacts (22.8%; 95% CI, 13.6%-33.5%).

**Conclusions and Relevance:**

The findings of this study suggest that given that individuals with suspected or confirmed infections are being referred to isolate at home, households will continue to be a significant venue for transmission of SARS-CoV-2.

## Key Points

**Question:**

What is the household secondary attack rate for severe acute respiratory syndrome coronavirus 2 (SARS-CoV-2)?

**Findings:**

In this meta-analysis of 54 studies with 77 758 participants, the estimated overall household secondary attack rate was 16.6%, higher than observed secondary attack rates for SARS-CoV and Middle East respiratory syndrome coronavirus. Controlling for differences across studies, secondary attack rates were higher in households from symptomatic index cases than asymptomatic index cases, to adult contacts than to child contacts, to spouses than to other family contacts, and in households with 1 contact than households with 3 or more contacts.

**Meaning:**

These findings suggest that households are and will continue to be important venues for transmission, even in areas where community transmission is reduced.

## Teaser

This systematic review and meta-analysis examines evidence for household transmission of severe acute respiratory syndrome coronavirus 2 (SARS-CoV-2), disaggregated by several covariates, and compares it with other coronaviruses

## Introduction

The coronavirus disease 2019 (COVID-19) pandemic is caused by severe acute respiratory syndrome coronavirus 2 (SARS-CoV-2), which is spread via direct or indirect contact with infected people via infected respiratory droplets or saliva, fomites, or aerosols.^1,2^ Crowded indoor environments with sustained close contact and conversations, such as households, are a particularly high-risk setting.^3^

The World Health Organization China Joint Mission reported human-to-human transmission in China largely occurred within families, accounting for 78% to 85% of clusters in Guangdong and Sichuan provinces.^4^ Stay-at-home orders reduced human mobility by 35% to 63% in the United States,^5^ 63% in the United Kingdom,^6^ and 54% in Wuhan,^7^ relative to normal conditions, which concomitantly increased time at home. Modeling studies demonstrated that household transmission had a greater relative contribution to the basic reproductive number after social distancing (30%-55%) than before social distancing (5%-35%).^8^ While current US Centers for Disease Control and Prevention recommendations are to maintain 6 feet of distance from a sick household member, this may be difficult to achieve in practice and not be fully effective.^9^

The household secondary attack rate characterizes virus transmissibility. Studies can collect detailed data on type, timing, and duration of contacts and identify risk factors associated with infectiousness of index cases and susceptibility of contacts. Our objective was to estimate the secondary attack rate of SARS-CoV-2 in households and determine factors that modify this parameter. We also estimated the proportion of households with index cases that had any secondary transmission. Furthermore, we compared the SARS-CoV-2 household secondary attack rate with that of other severe viruses and with that to close contacts for studies that reported the secondary attack rate for both close and household contacts.

## Methods

### Definitions

We estimated the transmissibility of SARS-CoV-2 within the household or family by the empirical secondary attack rate by dividing the number of new infections among contacts by the total number of contacts. Household contacts include anyone living in the same residence as the index case. Family contacts include the family members of index cases, including individuals who live outside the index case’s household. Close contact definitions varied by study and included physical proximity to an index case, exceeding a minimum contact time, and/or not wearing effective protection around index cases before the index case was tested.

### Search Strategy

Following Preferred Reporting Items for Systematic Reviews and Meta-analyses (PRISMA) reporting guideline, we searched PubMed using terms including *SARS-CoV-2* or *COVID-19* with *secondary attack rate*, *household*, *close contacts*, *contact transmission*, *contact attack rate*, or *family transmission* (eTable 1 in the Supplement) with no restrictions on language, study design, time, or place of publication. The last search was conducted October 19, 2020.

### Eligibility Criteria

Eligibility criteria are described in eAppendix 1 in the Supplement. All articles with original data for estimating household secondary attack rate were included. Case reports focusing on individual households and studies of close contacts that did not report secondary attack rates for household members were excluded.

### Data Extraction

One of us (Z.J.M.) extracted data from each study. Details appear in eAppendix 2 in the Supplement.

### Evaluation of Study Quality and Risk of Bias

To assess the methodological quality and risk of bias of included studies of SARS-CoV-2, we used the same modified version of the Newcastle-Ottawa quality assessment scale for observational studies used by Fung et al*.*^10,11^ Studies received as many as 9 points based on participant selection (4 points), study comparability (1 point), and outcome of interest (4 points). Studies were classified as having high (≤3 points), moderate (4-6 points), and low (≥7 points) risk of bias. One of us (Z.J.M.) evaluated the study quality and assigned the quality grades.

### Statistical Analysis

Meta-analyses were done using a restricted maximum-likelihood estimator model to yield Freeman-Tukey double arcsine–transformed point estimates and 95% CI for secondary attack rate for each subgroup analyzed, with a random effect for each study.^12^ For comparisons across covariates (ie, household or family, index case symptom status, adult or child contacts, contact sex, relationship to index case, adult or child index cases, index case sex, number of household contacts, study location, universal or symptomatic testing, dates of study) and comparisons with close contacts and other viruses, study was treated as a random effect, and the covariate was a fixed moderator. Variables had to have been collected in at least 3 studies to be included in meta-analyses. The Cochran Q test and *I*^2^ statistic are reported as measures of heterogeneity. *I*^2^ values of 25%, 50%, and 75% indicated low, moderate, and high heterogeneity, respectively.^13^ Stastistical significance was set at a 2-tailed α = .05. All analyses were done in R version 4.0.2 using the package metafor (R Project for Statistical Computing).^14,15^

When at least 10 studies were available, we used funnel plots, Begg correlation, and Egger test to evaluate publication bias, with significance set at *P* < .10.^16,17^ If we detected publication bias, we used the Duval and Tweedie trim-and-fill approach for adjustment.^18^

## Results

We identified 54 relevant published studies that reported household secondary transmission, with 77 758 participants (eTable 1 in the Supplement).^19,20,21,22,23,24,25,26,27,28,29,30,31,32,33,34,35,36,37,38,39,40,41,42,43,44,45,46,47,48,49,50,51,52,53,54,55,56,57,58,59,60,61,62,63,64,65,66,67,68,69,70,71,72^ A total of 16 of 54 studies (29.6%) were at high risk of bias, 27 (50.0%) were moderate, and 11 (20.4%) were low (eTable 2 in the Supplement). Lower quality was attributed to studies with 1 or fewer test per contact (35 studies [64.8%]), small sample sizes (31 [57.4%]), and secondary attack rate not disaggregated by covariates (28 [51.9%]).

A description of index case identification period and methods and symptom status is provided in eTable 3 in the Supplement. Most studies did not describe how co–primary index cases were handled or whether secondary infections could have been acquired from outside the household, both of which can inflate the empirical secondary attack rate. Testing and monitoring strategies varied between studies, often reflecting variations in local testing guidelines implemented as part of contact tracing (eTable 4 and eAppendix 3 in the Supplement).

**Figure 1.**
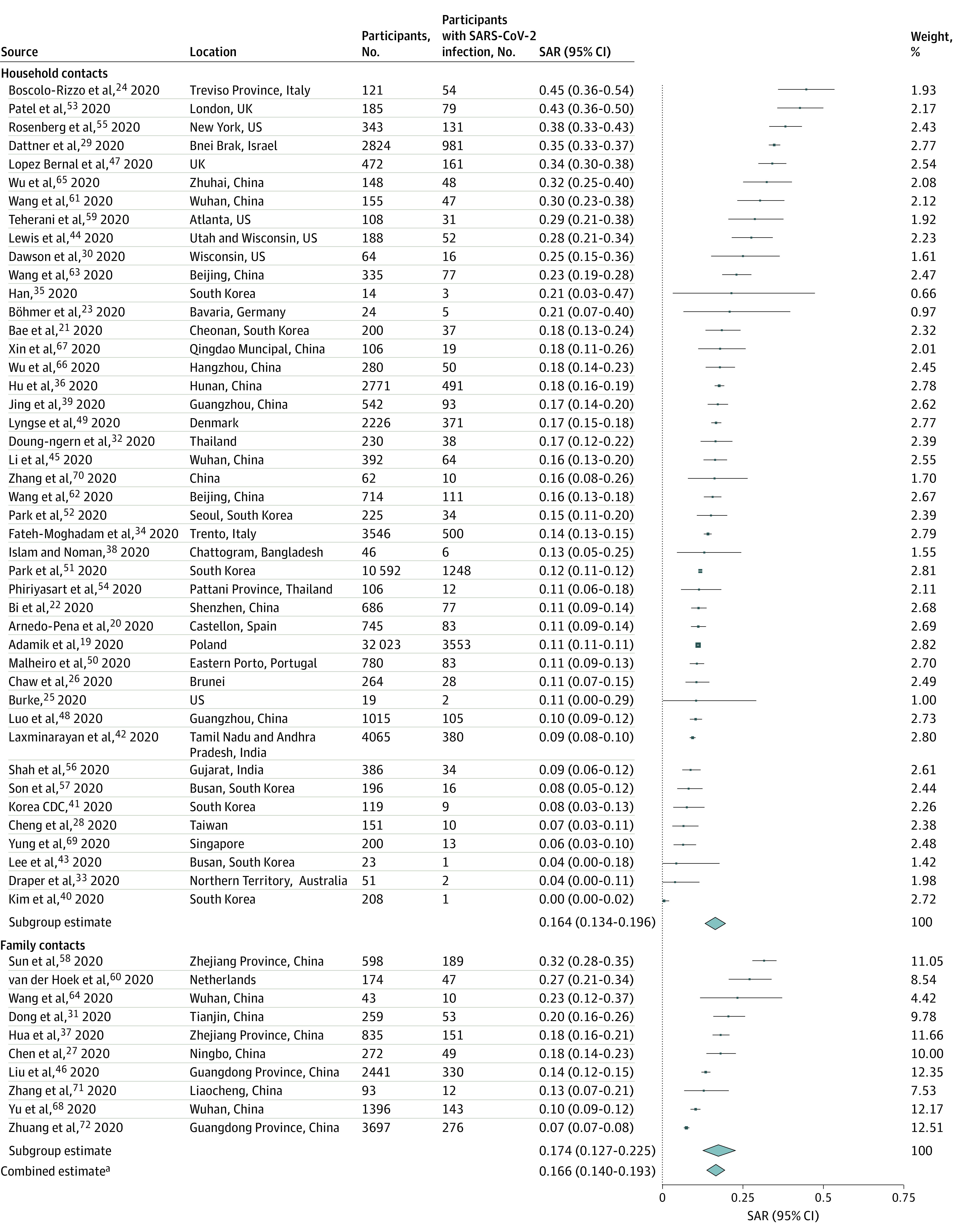
Secondary Attack Rates (SAR) of Severe Acute Respiratory Syndrome Coronavirus 2 (SARS-CoV-2) for Household Contacts and Family Contacts Point sizes are an inverse function of the precision of the estimates, and bars correspond to 95% CIs. CDC indicates Centers for Disease Control and Prevention. ^a^Weights for the combined estimate are available in eTable 8 in the Supplement.

Figure 1 summarizes secondary attack rates for 44 studies^19,20,21,22,23,24,25,26,28,29,30,32,33,34,35,36,38,39,40,41,42,43,44,45,47,48,49,50,51,52,53,54,55,56,57,59,61,62,63,65,66,67,69,70^ of household contacts and 10 of family contacts.^26,31,37,45,58,60,65,68,71,72^ Estimated mean secondary attack rate for household contacts was 16.4% (95% CI, 13.4%-19.6%) and family contacts was 17.4% (95% CI, 12.7%-22.5%). One study^40^ restricted index cases to children (age <18 years), resulting in a substantially lower secondary attack rate of 0.5%. Excluding this outlier, the combined secondary attack rate for household and family contacts was 17.1% (95%, 14.6%-19.7%). Secondary attack rates for household and family contacts were more than 3 times higher than for close contacts (4.8%; 95% CI, 3.4%-6.5%; *P* < .001) (eFigure 2 in the Supplement). Significant heterogeneity was found among studies of household (*I^2^* = 96.9%; *P* < .001), family (*I^2^* = 93.0%; *P* < .001), and close (*I^2^* = 97.0%; *P* < .001) contacts. No significant publication bias was observed for studies of household, family, or close contacts (eFigure 3 in the Supplement). Secondary attack rates were not significantly different when restricting to 38 studies^19,20,22,23,26,27,28,29,30,31,34,35,36,37,38,39,40,42,44,45,46,47,48,49,50,51,54,55,56,57,60,62,63,65,67,68,69,72^ with low or moderate risk of bias (15.6%; 95%, 12.8%-18.5%) (eFigure 4 in the Supplement). There were no significant differences in secondary attack rates between 21 studies in China^22,27,31,36,37,39,45,46,48,58,61,62,63,64,65,66,67,68,70,71,72^ and 33 studies from other countries^19,20,21,23,24,25,26,28,29,30,32,33,34,35,38,40,41,42,43,44,47,49,50,51,52,53,54,55,56,57,59,60,69^ (eFigure 5 in the Supplement), 18 studies that tested symptomatic contacts^19,20,21,24,25,28,29,33,34,41,47,50,53,56,58,59,61,64^ and 33 studies that reported testing all contacts^22,23,26,27,30,31,35,36,37,38,39,40,42,43,44,45,46,48,49,51,52,54,55,57,60,63,65,66,67,69,70,71,72^ (eFigure 6 in the Supplement), and 16 early studies^22,23,25,31,37,39,45,58,61,63,64,65,66,68,71,72^ (January-February) and 20 later studies^19,24,26,29,30,32,33,34,35,38,42,44,50,53,54,55,56,59,60,69^ (March-July) (eFigure 7 in the Supplement).

To study the transmissibility of asymptomatic SARS-CoV-2 index cases, eFigure 8 in the Supplement summarizes 27 studies^19,20,21,23,24,25,26,30,32,33,34,44,45,47,50,52,53,54,56,59,60,61,63,64,68,69,72^ reporting household secondary attack rates from symptomatic index cases and 4 studies^26,43,44,52^ from asymptomatic or presymptomatic index cases. Estimated mean household secondary attack rate from symptomatic index cases (18.0%; 95% CI, 14.2%-22.1%) was significantly higher than from asymptomatic or presymptomatic index cases (0.7%; 95% CI, 0%-4.9%; *P* < .001), although there were few studies in the latter group. These findings are consistent with other household studies^28,70^ reporting asymptomatic index cases as having limited role in household transmission.

**Figure 2.**
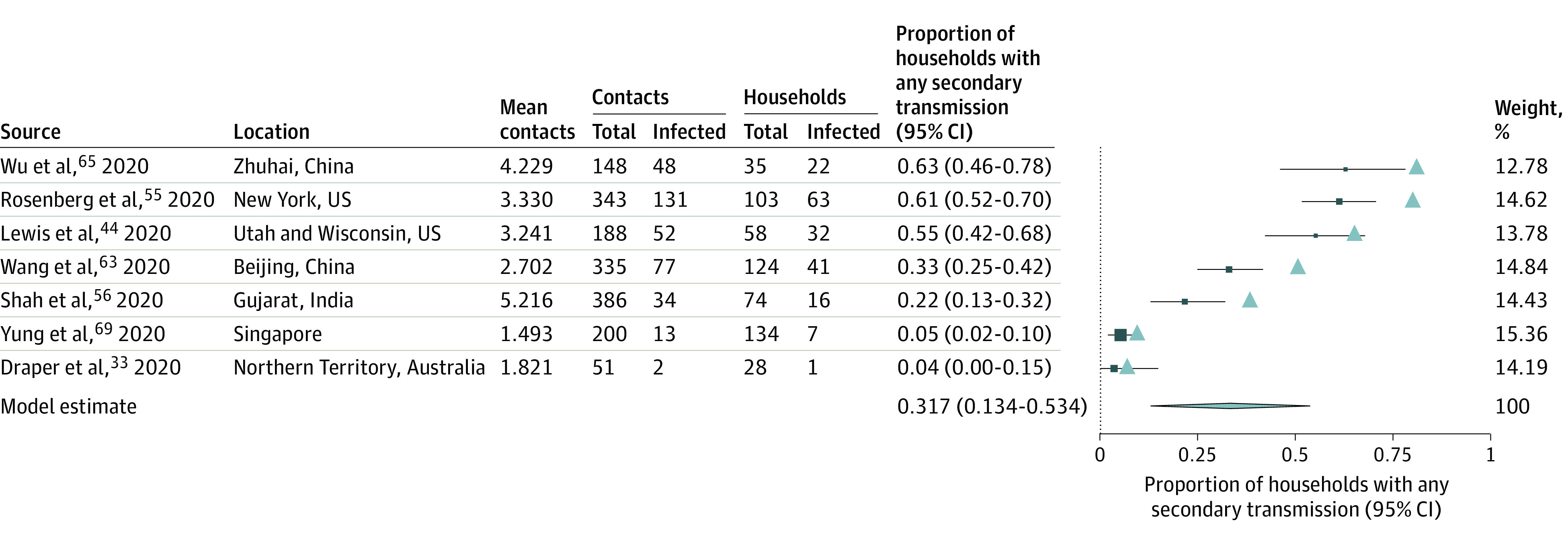
Mean Number of Contacts per Household, Secondary Attack Rate (SAR) of Severe Acute Respiratory Syndrome Coronavirus 2 (SARS-CoV-2), and Proportion of Households Reporting Any Secondary Transmission From Index Cases The expected proportion of households with any secondary transmission (represented by the triangles) was calculated as proportion with at least 1 secondary infection in a household = 1 − (1 −*SAR*)*^n^*, where *n* is the mean number of contacts for that study (eTable 5 in the Supplement). Point sizes are an inverse function of the precision of the estimates, and bars correspond to 95% CIs.

There is evidence for clustering of SARS-CoV-2 infections within households, with some households having many secondary infections while many others have none.^73,74,75^ For example, 1 study^55^ reported that 26 of 103 (25.2%) households had all members test positive. This is consistent with observation of overdispersion in the number of secondary cases per index case across a range of settings.^3^ While most studies reported only the average number of secondary infections per index case, some also reported transmission by household.^44,55,56,63,65,69^ Figure 2 summarizes the proportion of households with any secondary transmission. Using an empirical analysis based on secondary attack rates and mean number of contacts per household, we found the proportion of households with any secondary transmission was lower than expected in a setting with no clustering (eg, most transmission is not characterized by a minority of infected individuals) (eTable 5 in the Supplement). Ideally, future studies will assess this formally by fitting a β binomial to quantify overdispersion in the full data.

**Figure 3.**
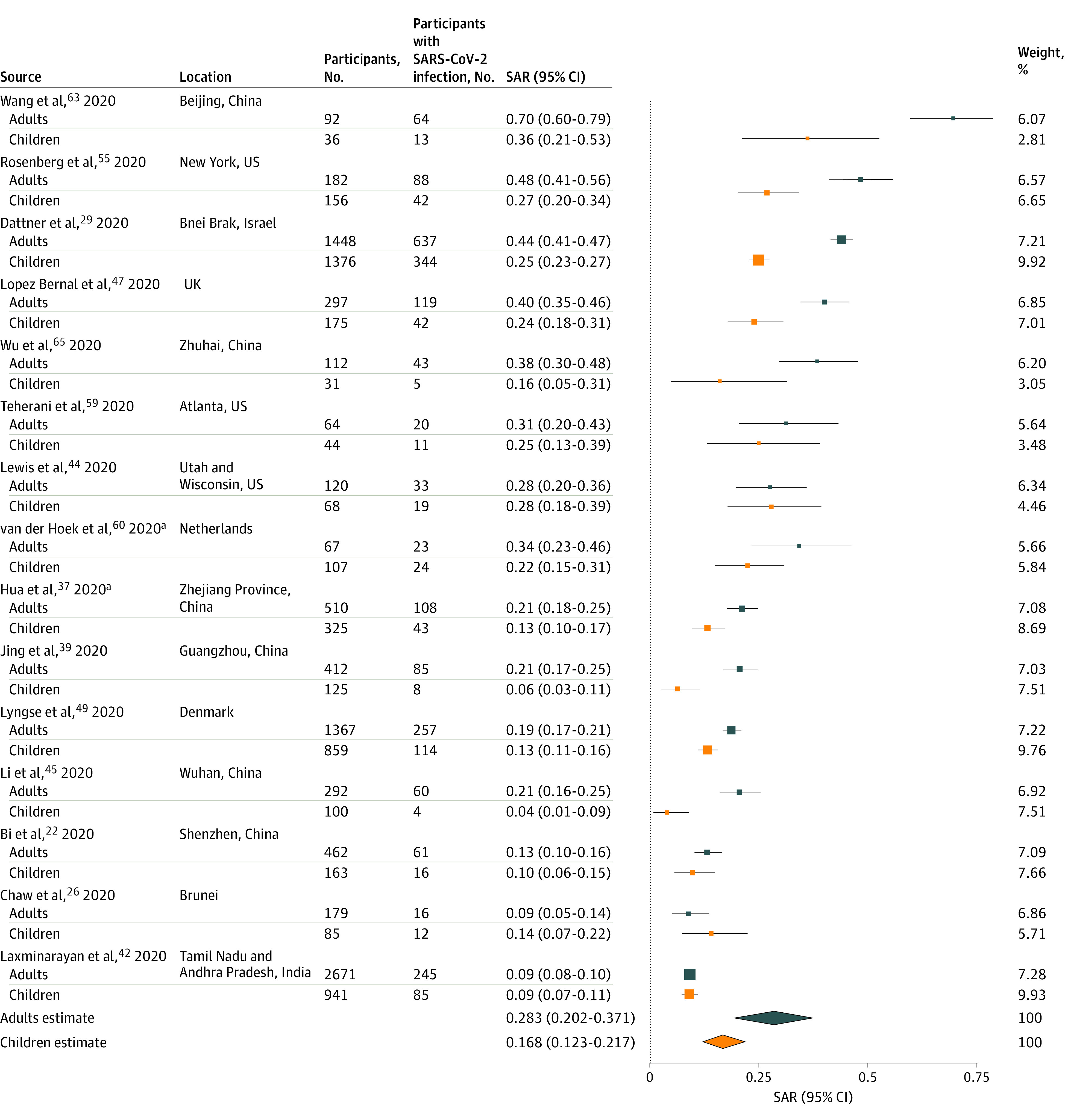
Secondary Attack Rates (SAR) of Severe Acute Respiratory Syndrome Coronavirus 2 (SARS-CoV-2) for Adult (≥18 Years) and Child (<18 Years) Household and Family Contacts Point sizes are an inverse function of the precision of the estimates and bars correspond to 95% CIs. ^a^Study of family contacts.

A number of studies examined factors associated with susceptibility of household contacts to infection (eTable 6 in the Supplement). Age was the most examined covariate, with most studies^20,29,36,37,38,39,45,46,48,49,55,63,65,68^ reporting lower secondary transmission of SARS-CoV-2 to child contacts than adult contacts. In 5 studies,^20,36,39,48,49^ individuals older than 60 years were most susceptible to SARS-CoV-2 infection. Contact age was not associated with susceptibility in 9 studies,^26,28,32,44,47,58,66,67,70^ although these were typically less powered to detect a difference. Figure 3 summarizes 15 studies^22,26,29,37,39,42,44,45,47,49,55,59,60,63,65^ reporting separate secondary attack rates to children and adult contacts. The estimated mean household secondary attack rate was significantly higher to adult contacts (28.3%; 95% CI, 20.2%-37.1%) than to child contacts (16.8%; 95% CI, 12.3%-21.7%; *P* < .001). Significant heterogeneity was found among studies of adult (*I^2^* = 96.8%; *P* < .001) and child contacts (*I^2^* = 78.9%; *P* < .001). Begg (*P* = .03) and Egger (*P* = .03) tests were statistically significant for studies of adult but not child contacts (eFigure 9 in the Supplement). One study of adults^63^ had a high secondary attack rate in the forest plot. Excluding this study improved the funnel plot symmetry and resulted in a secondary attack rate to adult contacts of 26.3% (95% CI, 19.3%-33.2%).

The second most examined factor was sex of exposed contacts, which was not associated with susceptibility for most studies^20,22,26,32,36,39,44,45,47,48,49,58,65,66,67,70^ except 3.^38,46,68^ eFigure 10 in the Supplement summarizes results from 11 studies^20,39,42,44,45,47,49,58,65,67,69^ reporting household secondary attack rates by contact sex. Estimated mean household secondary attack rate to female contacts (20.7%; 95% CI, 15.0%-26.9%) was not significantly different than to male contacts (17.7%; 95% CI, 12.4%-23.8%). Significant heterogeneity was found among studies of female contacts (*I^2^* = 87.4%; *P* < .001) and male contacts (*I^2^* = 87.7%; *P* < .001). Moderate asymmetry was observed in the funnel plots, which was significant for studies of female contacts from Egger test (*P* = .07) but not male contacts (eFigure 11 in the Supplement). However, imputation of an adjusted effect size using the trim-and-fill method did not significantly change the secondary attack rate to female contacts (19.7%; 95% CI, 13.9%-25.6%).

**Figure 4.**
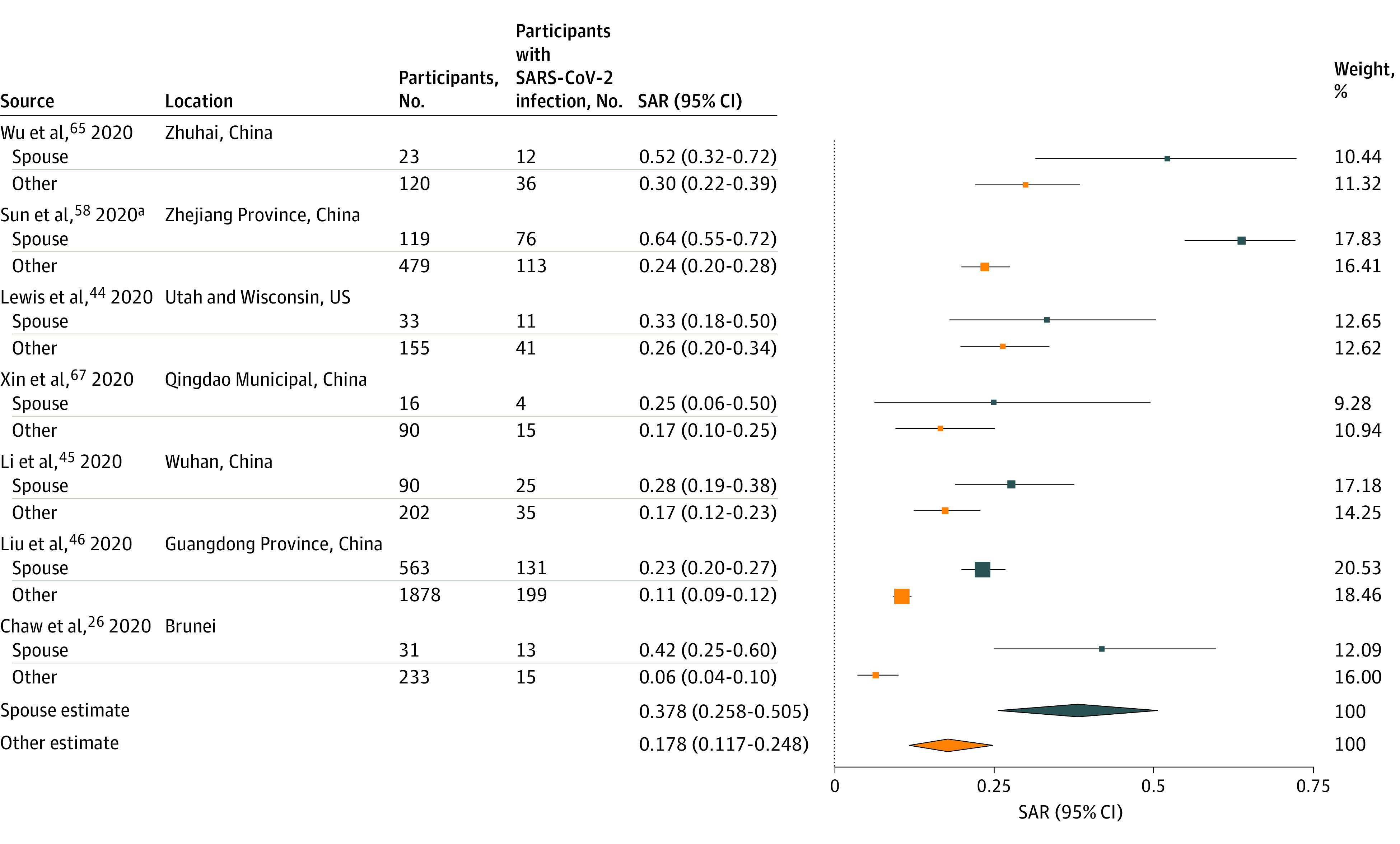
Secondary Attack Rates (SAR) of Severe Acute Respiratory Syndrome Coronavirus 2 (SARS-CoV-2) for Household and Family Contacts by Relationship to Index Case Point sizes are an inverse function of the precision of the estimates and bars correspond to 95% CIs. ^a^Study of family contacts.

Spouse relationship to index case was associated with secondary infection in 4 studies^26,45,46,58^ of 6 in which this was examined.^65,67^ Infection risk was highest for spouses, followed by nonspouse family members and other relatives, which were all higher than other contacts.^46^ Figure 4 summarizes results from 7 studies^26,44,45,46,58,65,67^ reporting household secondary attack rates by relationship. Estimated mean household secondary attack rate to spouses (37.8%; 95% CI, 25.8%-50.5%) was significantly higher than to other contacts (17.8%; 95% CI, 11.7%-24.8%). Significant heterogeneity was found among studies of spouses (*I^2^* = 78.6%; *P* < .001) and other relationships (*I^2^* = 83.5%; *P* < .001).

Several studies examined factors associated with infectiousness of index cases. Older index case age was associated with increased secondary infections in 3 studies^20,47,67^ of 9 in which this was examined.^22,36,39,44,63,65^ eFigure 12 in the Supplement summarizes results from 3 studies^42,44,51^ reporting household secondary attack rates by index case age. Estimated mean household secondary attack rate from adults (15.2%; 95% CI, 6.2%-27.4%) was not significantly different than that from children (7.9%; 95% CI, 1.7%-16.8%). Index case sex was associated with transmission in 3 studies^42,44,67^ of 9 in which this was examined.^20,36,45,47,63,65^ eFigure 13 in the Supplement summarizes results from 7 studies^20,42,44,45,65,67,69^ reporting household secondary attack rates by index case sex. Estimated mean household secondary attack rate from female contacts (16.6%; 95% CI, 11.2%-22.8%) was not significantly different than from male contacts (16.4%; 95% CI, 9.0%-25.5%).

Critically severe index case symptoms was associated with higher infectiousness in 6 studies^20,38,46,47,48,67^ of 9 in which this was examined.^44,63,70^ Index case cough was associated with infectivity in 2 studies ^20,65^ of 8 in which this was examined^45,46,47,48,63,67^ (eAppendix 4 in the Supplement).

**Figure 5.**
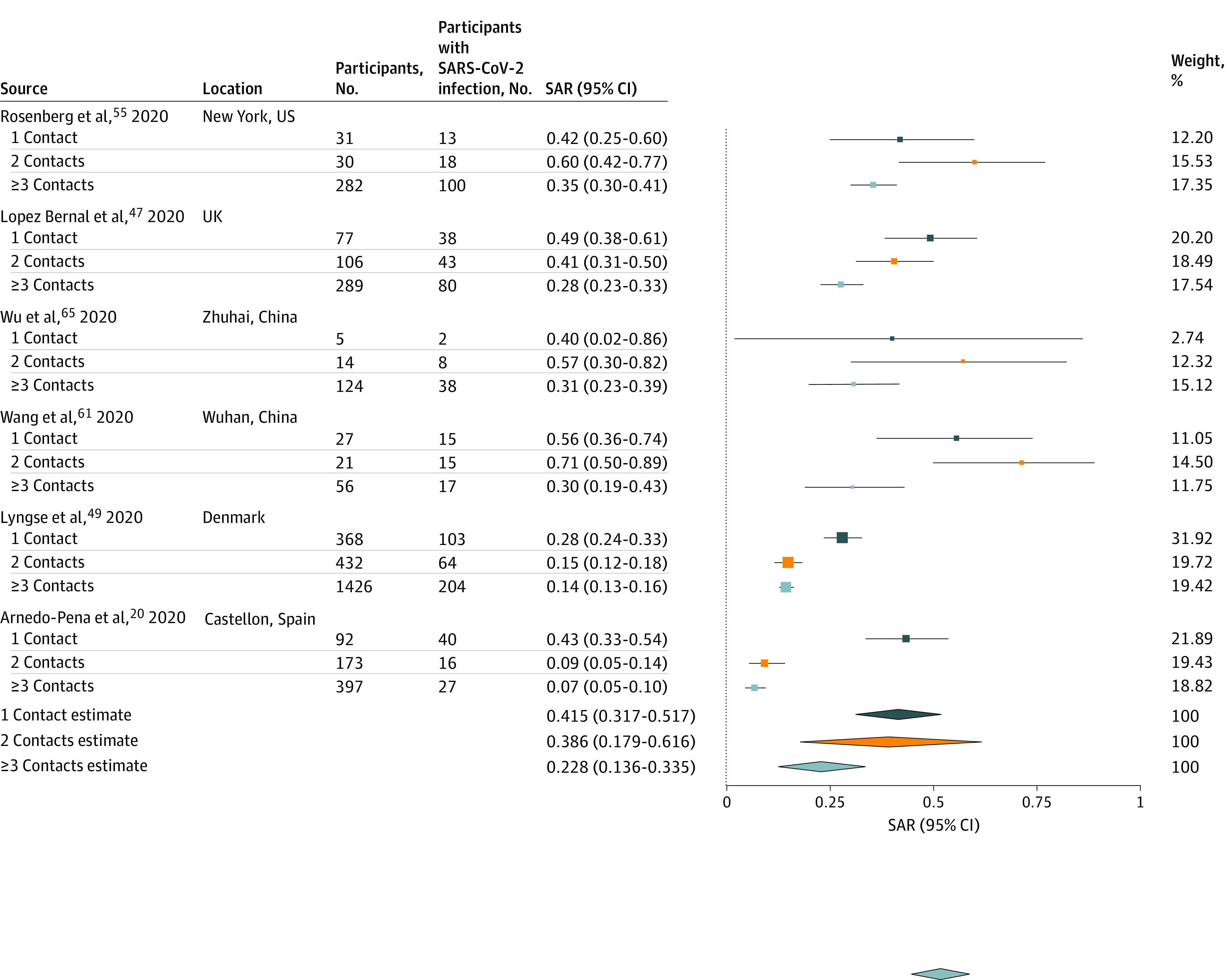
Secondary Attack Rates (SAR) of Severe Acute Respiratory Syndrome Coronavirus 2 (SARS-CoV-2) by the Number of People Living in the Same Household as the Index Case Point sizes are an inverse function of the precision of the estimates, and bars correspond to 95% CIs.

Contact frequency with the index case was associated with higher odds of infection, specifically at least 5 contacts during 2 days before the index case was confirmed,^70^ at least 4 contacts and 1 to 3 contacts,^63^ or frequent contact within 1 meter.^22,67,68^ Smaller households were associated with transmission in 4 studies^20,39,47,49^ of 7 in which this was examined.^55,63,65^ Figure 5 summarizes results from 6 studies^20,47,49,55,61,65^ reporting household secondary attack rates by number of contacts in the household. Estimated mean household secondary attack rate for households with 1 contact (41.5%; 95% CI, 31.7%-51.7%) was significantly higher than households with at least 3 contacts (22.8%; 95% CI, 13.6%-33.5%; *P* < .001) but not different than households with 2 contacts (38.6%; 95% CI, 17.9%-61.6%). There was significant heterogeneity in secondary attack rates between studies with 1 contact (*I^2^* = 52.9%; *P* = .049), 2 contacts (*I^2^* = 93.6%; *P* < .001), or 3 or more contacts (*I^2^* = 91.6%; *P* < .001). Information was not available on household crowding (eg, number of people per room).

eFigure 14 in the Supplement summarizes 7 studies^76,77,78,79,80,81,82^ reporting household secondary attack rates for SARS-CoV, and 7 studies^83,84,85,86,87,88,89^ for Middle East respiratory syndrome coronavirus (MERS-CoV). Estimated mean household secondary attack rate was 7.5% (95% CI, 4.8%-10.7%) for SARS-CoV and 4.7% (95% CI, 0.9%-10.7%) for MERS-CoV (eTable 7 in the Supplement), both lower than the household secondary attack rate of 16.6% for SARS-CoV-2 in this study (*P* < .001). The SARS-CoV-2 secondary attack rate was also higher than secondary attack rates reported for HCoV-NL63 (0-12.6%), HCoV-OC43 (10.6-13.2%), HCoV-229E (7.2-14.9%), and HCoV-HKU1 (8.6%).^90,91,92^ Household secondary attack rates for SARS-CoV-2 were within the mid-range of household secondary attack rates reported for influenza, which ranged from 1% to 38% based on polymerase chain reaction–confirmed infection.^93^

## Discussion

We synthesized the available evidence on household studies of SARS-CoV-2. The combined household and family secondary attack rate was 16.6% (95% CI, 14.0%-19.3%), although with significant heterogeneity between studies. This point estimate is higher than previously observed secondary attack rates for SARS-CoV and MERS-CoV. Households are favorable environments for transmission. They are what are known as 3Cs environments, as they are closed spaces, where family members may crowd and be in close contact with conversation.^94^ There may be reduced use of personal protective equipment relative to other settings.

That secondary attack rates were not significantly different between household and family contacts may indicate that most family contacts are in the same household as index cases. Household and family contacts are at higher risk than other types of close contacts, and risks are not equal within households. Spouses were at higher risk than other family contacts, which may explain why the secondary attack rate was higher in households with 1 vs 3 or greater contacts. Spouse relationship to the index case was also a significant risk factor observed in studies of SARS-CoV and H1N1.^82,95^ This may reflect intimacy, sleeping in the same room, or longer or more direct exposure to index cases. Further investigation is required to determine whether sexual contact is a transmission route. Although not directly assessed, household crowding (eg, number of people per room) may be more important for SARS-CoV-2 transmission than the total number of people per household, as has been demonstrated for influenza.^96,97,98^

The finding that secondary attack rates were higher to adult contacts than to child contacts is consistent with empirical and modeling studies.^99,100^ Lower infection rates in children may be attributed to asymptomatic or mild disease, reduced susceptibility from cross-immunity from other coronaviruses,^101^ and low case ascertainment,^102^ but the difference persisted in studies in which all contacts were tested regardless of symptoms. Higher transmission rates to adults may be influenced by spousal transmission. Given the increased risk to spousal contacts, future studies might compare child contacts and nonspouse adult contacts to ascertain whether this difference persists. Limited data suggest children have not played a substantive role in household transmission of SARS-CoV-2.^40,103,104,105^ However, a study in South Korea of 10 592 household contacts noted relatively high transmission from index cases who were aged 10 to 19 years.^51^ Although children seem to be at reduced risk for symptomatic disease, it is still unclear whether they shed virus similarly to adults.^106^

We did not find associations between household contact or index case sex and secondary transmission. The World Health Organization reports roughly even distribution of SARS-CoV-2 infections between women and men worldwide, with higher mortality in men.^107^

We found significantly higher secondary attack rates from symptomatic index cases than asymptomatic or presymptomatic index cases, although less data were available on the latter. The lack of substantial transmission from observed asymptomatic index cases is notable. However, presymptomatic transmission does occur, with some studies reporting the timing of peak infectiousness at approximately the period of symptom onset.^108,109^ In countries where infected individuals were isolated outside the home, this could further alter the timing of secondary infections by limiting contacts after illness onset.^110^

Household secondary attack rates were higher for SARS-CoV-2 than SARS-CoV and MERS-CoV, which may be attributed to structural differences in spike proteins,^111^ higher basic reproductive rates,^112^ and higher viral loads in the nose and throat at the time of symptom onset.^113^ Symptoms associated with MERS-CoV and SARS-CoV often require hospitalization, which increases nosocomial transmission, whereas less severe symptoms of SARS-CoV-2 facilitate community transmission.^113^ Similarly, presymptomatic transmission was not observed for MERS-CoV or SARS-CoV.^114,115^

### Limitations

Our study had several limitations. The most notable is the large amount of unexplained heterogeneity across studies. This is likely attributable to variability in study definitions of index cases and household contacts, frequency and type of testing, sociodemographic factors, household characteristics (eg, density, air ventilation), and local policies (eg, centralized isolation). Rates of community transmission also varied across locations. Given that studies cannot always rule out infections from outside of the home (eg, nonhousehold contacts), household transmission may be overestimated. For this reason, we excluded studies that used antibody tests to diagnose household contacts. Furthermore, many analyses ignored tertiary transmission within the household, classifying all subsequent cases as secondary to the index case. Eighteen studies^19,20,21,24,25,28,29,33,34,41,47,50,53,56,58,59,61,64^ involved testing only symptomatic household contacts, which would miss asymptomatic or subclinical infections, although secondary attack rate estimates were similar across studies testing all vs only symptomatic contacts.

Important questions remain regarding household spread of SARS-CoV-2. Chief among them is the infectiousness of children to their household contacts and the infectiousness of asymptomatic, mildly ill, and severely ill index cases. This study did not provide additional elucidation of factors influencing intergenerational spread. People unable to work at home may have greater risk of SARS-CoV-2 exposure, which may increase transmission risk to other household members. There may be overdispersion in the number of secondary infections per index case, which could be caused by variations in viral shedding, household ventilation, or other factors.

## Conclusions

The findings of this study suggest that households are and will continue to be important venues for transmission, even where community transmission is reduced. Prevention strategies, such as increased mask-wearing at home, improved ventilation, voluntary isolation at external facilities, and targeted antiviral prophylaxis, should be further explored.

## Notes

Supplement. **eFigure 1. **PRISMA Flow Diagram for Review of Household Secondary Attack of SARS-CoV-2, MERS-CoV, SARS-CoV, and Other Coronaviruses **eFigure 2. **Secondary Attack Rates of SARS-CoV-2 for Studies of Close Contacts **eFigure 3. **Funnel Plots of Studies Reporting Secondary Attack Rates of SARS-CoV-2 for Household, Family, and Close Contacts **eFigure 4. **Household Secondary Attack Rates of SARS-CoV-2, Restricted to Studies With Low or Moderate Risk of Bias as Determined by the Modified Newcastle-Ottawa Scale **eFigure 5. **Household Secondary Attack Rates of SARS-CoV-2, Grouped by Studies in China vs Other Locations **eFigure 6. **Secondary Attack Rates of SARS-CoV-2, Grouped by Studies That Tested Only Symptomatic Household Contacts and Studies That Tested All Household Contacts Irrespective of Symptoms **eFigure 7. **Household Secondary Attack Rates of SARS-CoV-2, Grouped by Studies Early (January-February) and Later (March-July) in the Pandemic **eFigure 8. **Secondary Attack Rates of SARS-CoV-2 From Symptomatic and Asymptomatic or Presymptomatic Index Cases to Household and Family Contacts **eFigure 9. **Funnel Plots of Studies Reporting Household Secondary Attack Rates of SARS-CoV-2 for Adult (≥18 Years) and Child (<18 Years) Contacts **eFigure 10. **Secondary Attack Rates of SARS-CoV-2 for Household and Family Contacts by Contact Sex **eFigure 11. **Funnel Plots of Studies Reporting Household Secondary Attack Rates of SARS-CoV-2 for Female and Male Contacts **eFigure 12. **Secondary Attack Rates of SARS-CoV-2 to Household Contacts From Adult (≥18 Years) and Child (<18 Years) Index Cases **eFigure 13. **Secondary Attack Rates of SARS-CoV-2 for Household Contacts by Index Case Sex **eFigure 14. **Household Secondary Attack Rates of SARS-CoV and MERS-CoV **eTable 1. **Electronic Databases and Search Strategy for Household Secondary Attack Rate of SARS-CoV-2, MERS-CoV, SARS-CoV, and Other Coronaviruses **eTable 2. **Risk of Bias Assessment for Studies Included in Review of Household Transmissibility of SARS-CoV-2 **eTable 3. **Description of Index Cases for Studies Included in Review of Household Transmissibility of SARS-CoV-2 **eTable 4. **Description of Contacts for Studies Included in Review of Household Transmissibility of SARS-CoV-2 **eTable 5. **Overdispersion of the Number of Secondary Infections of SARS-CoV-2 per Household **eTable 6. **Assessment of Factors Potentially Affecting Susceptibility and Infectivity of SARS-CoV-2 in Household Transmission Studies **eTable 7. **Household Secondary Attack Rate Comparison With Other Viruses **eTable 8. **Weights for Combined Estimate of Secondary Attack Rates of Severe Acute Respiratory Syndrome Coronavirus 2 (SARS-CoV-2) for Household Contacts and Family Contacts **eAppendix 1. **Eligibility Criteria **eAppendix 2. **Data Extraction **eAppendix 3. **Additional Description of Studies **eAppendix 4. **Additional Description of Risk Factors **eReferences.**
