# Supplementary material for: Household Transmission of SARS-CoV-2: A Systematic Review and Meta-analysis
Source: JAMA Netw Open. 2020 Dec 14;3(12):e2031756. doi: 10.1001/jamanetworkopen.2020.31756 (PMC7737089; doi:10.1001/jamanetworkopen.2020.31756)
Supplement: Supplement. — eFigure 1. PRISMA Flow Diagram for Review of Household Secondary Attack of SARS-CoV-2, MERS-CoV, SARS-CoV, and Other Coronaviruses eFigure 2. Secondary Attack Rates of SARS-CoV-2 for Studies of Close Contacts eFigure 3. Funnel Plots of Studies Reporting Secondary Attack Rates of SARS-CoV-2 for Household, Family, and Close Contacts eFigure 4. Household Secondary Attack Rates of SARS-CoV-2, Restricted to Studies With Low or Moderate Risk of Bias as Determined by the Modified Newcastle-Ottawa Scale eFigure 5. Household Secondary Attack Rates of SARS-CoV-2, Grouped by Studies in China vs Other Locations eFigure 6. Secondary Attack Rates of SARS-CoV-2, Grouped by Studies That Tested Only Symptomatic Household Contacts and Studies That Tested All Household Contacts Irrespective of Symptoms eFigure 7. Household Secondary Attack Rates of SARS-CoV-2, Grouped by Studies Early (January-February) and Later (March-July) in the Pandemic eFigure 8. Secondary Attack Rates of SARS-CoV-2 From Symptomatic and Asymptomatic or Presymptomatic Index Cases to Household and Family Contacts eFigure 9. Funnel Plots of Studies Reporting Household Secondary Attack Rates of SARS-CoV-2 for Adult (≥18 Years) and Child (<18 Years) Contacts eFigure 10. Secondary Attack Rates of SARS-CoV-2 for Household and Family Contacts by Contact Sex eFigure 11. Funnel Plots of Studies Reporting Household Secondary Attack Rates of SARS-CoV-2 for Female and Male Contacts eFigure 12. Secondary Attack Rates of SARS-CoV-2 to Household Contacts From Adult (≥18 Years) and Child (<18 Years) Index Cases eFigure 13. Secondary Attack Rates of SARS-CoV-2 for Household Contacts by Index Case Sex eFigure 14. Household Secondary Attack Rates of SARS-CoV and MERS-CoV eTable 1. Electronic Databases and Search Strategy for Household Secondary Attack Rate of SARS-CoV-2, MERS-CoV, SARS-CoV, and Other Coronaviruses eTable 2. Risk of Bias Assessment for Studies Included in Review of Household Transmissibility of SARS-CoV-2 eTable 3. [file jamanetwopen-e2031756-s001.pdf]

Supplemental Online Content

Madewell ZJ, Yang Y, Longini IM Jr, Halloran ME, Dean NE. Household transmission of SARS-CoV-2: a systematic review and meta-analysis. *JAMA Netw Open*. 2020;3(12):e2031756. doi:10.1001/jamanetworkopen.2020.31756

eFigure 1. PRISMA Flow Diagram for Review of Household Secondary Attack of SARS-CoV-2, MERS-CoV, SARS-CoV, and Other Coronaviruses

eFigure 2. Secondary Attack Rates of SARS-CoV-2 for Studies of Close Contacts

eFigure 3. Funnel Plots of Studies Reporting Secondary Attack Rates of SARS-CoV-2 for Household, Family, and Close Contacts

eFigure 4. Household Secondary Attack Rates of SARS-CoV-2, Restricted to Studies With Low or Moderate Risk of Bias as Determined by the Modified Newcastle-Ottawa Scale

eFigure 5. Household Secondary Attack Rates of SARS-CoV-2, Grouped by Studies in China vs Other Locations

eFigure 6. Secondary Attack Rates of SARS-CoV-2, Grouped by Studies That Tested Only Symptomatic Household Contacts and Studies That Tested All Household Contacts Irrespective of Symptoms

eFigure 7. Household Secondary Attack Rates of SARS-CoV-2, Grouped by Studies Early (January-February) and Later (March-July) in the Pandemic

eFigure 8. Secondary Attack Rates of SARS-CoV-2 From Symptomatic and Asymptomatic or Presymptomatic Index Cases to Household and Family Contacts

eFigure 9. Funnel Plots of Studies Reporting Household Secondary Attack Rates of SARS-CoV-2 for Adult (≥18 Years) and Child (<18 Years) Contacts

eFigure 10. Secondary Attack Rates of SARS-CoV-2 for Household and Family Contacts by Contact Sex

eFigure 11. Funnel Plots of Studies Reporting Household Secondary Attack Rates of SARS-CoV-2 for Female and Male Contacts

eFigure 12. Secondary Attack Rates of SARS-CoV-2 to Household Contacts From Adult (≥18 Years) and Child (<18 Years) Index Cases

eFigure 13. Secondary Attack Rates of SARS-CoV-2 for Household Contacts by Index Case Sex

eFigure 14. Household Secondary Attack Rates of SARS-CoV and MERS-CoV

© 2020 Madewell ZJ et al. *JAMA Network Open.*

eTable 1. Electronic Databases and Search Strategy for Household Secondary Attack Rate of SARS-CoV-2, MERS-CoV, SARS-CoV, and Other Coronaviruses

eTable 2. Risk of Bias Assessment for Studies Included in Review of Household Transmissibility of SARS-CoV-2

eTable 3. Description of Index Cases for Studies Included in Review of Household Transmissibility of SARS-CoV-2

eTable 4. Description of Contacts for Studies Included in Review of Household Transmissibility of SARS-CoV-2

eTable 5. Overdispersion of the Number of Secondary Infections of SARS-CoV-2 per Household

eTable 6. Assessment of Factors Potentially Affecting Susceptibility and Infectivity of SARS-CoV-2 in Household Transmission Studies

eTable 7. Household Secondary Attack Rate Comparison With Other Viruses

eTable 8. Weights for Combined Estimate of Secondary Attack Rates of Severe Acute Respiratory Syndrome Coronavirus 2 (SARS-CoV-2) for Household Contacts and Family Contacts

eAppendix 1. Eligibility Criteria

eAppendix 2. Data Extraction

eAppendix 3. Additional Description of Studies

eAppendix 4. Additional Description of Risk Factors

eReferences.

This supplemental material has been provided by the authors to give readers additional information about their work.

© 2020 Madewell ZJ et al. *JAMA Network Open.*

eFigure 1. PRISMA Flow Diagram for Review of Household Secondary Attack of SARS-CoV-2, MERS-CoV, SARS-CoV, and Other Coronaviruses

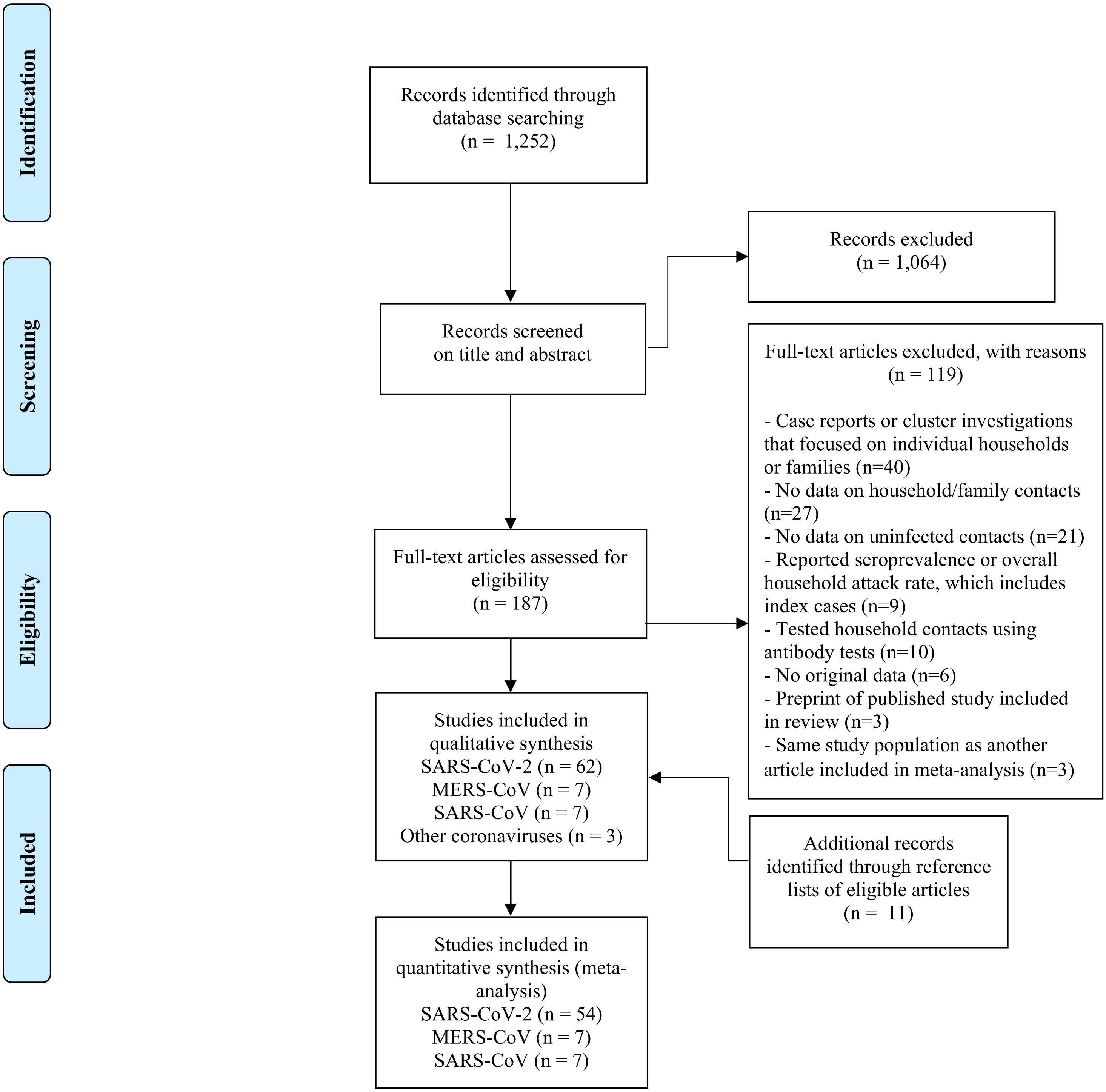

© 2020 Madewell ZJ et al. *JAMA Network Open.*

eFigure 2. Secondary Attack Rates of SARS-CoV-2 for Studies of Close Contacts

Author, Location	Infected	Total	SAR (95% CI)
Han et al., South Korea	7	36	0.19 [0.08, 0.34]
Laxminarayan et al., TN & AP, India	623	4637	0.13 [0.12, 0.14]
Fateh-Moghadam et al., Trento, Italy	840	6255	0.13 [0.13, 0.14]
Malheiro et al., Eastern Porto, Portugal	154	1627	0.09 [0.08, 0.11]
Yu et al., Wuhan, China	150	1587	0.09 [0.08, 0.11]
Bi et al., Shenzhen, China	98	1286	0.08 [0.06, 0.09]
Böhmer et al., Bavaria, Germany	16	241	0.07 [0.04, 0.10]
Phiriyasart et al., Pattani Province, Thailand	25	387	0.06 [0.04, 0.09]
Bae et al., Cheonan, Korea	108	1687	0.06 [0.05, 0.08]
Chen et al., Ningbo, China	132	2147	0.06 [0.05, 0.07]
Wang, Pan et al., Beijing, China	186	4007	0.05 [0.04, 0.05]
Liu et al., Guangdong Province, China	515	11580	0.04 [0.04, 0.05]
Luo et al., Guangzhou, China	127	3410	0.04 [0.03, 0.04]
Park, Choe et al., South Korea	2169	59073	0.04 [0.04, 0.04]
Islam et al., Chattogram, Bangladesh	13	391	0.03 [0.02, 0.05]
Zhang, Cheng et al., China	12	369	0.03 [0.02, 0.05]
Hu et al., Hunan, China	471	15648	0.03 [0.03, 0.03]
Chaw et al., Brunei	51	1755	0.03 [0.02, 0.04]
Zhuang et al., Guangdong Province, China	239	8319	0.03 [0.03, 0.03]
Wu, Song et al., Hangzhou, China	82	2994	0.03 [0.02, 0.03]
Draper et al., Northern Territory, Australia	4	445	0.01 [0.00, 0.02]
Cheng et al., Taiwan	22	2761	0.01 [0.00, 0.01]
Korea CDC, South Korea	13	2370	0.01 [0.00, 0.01]
Burke et al. , USA	2	445	0.00 [0.00, 0.01]
Model Estimate	0.048 (0.034, 0.065)		

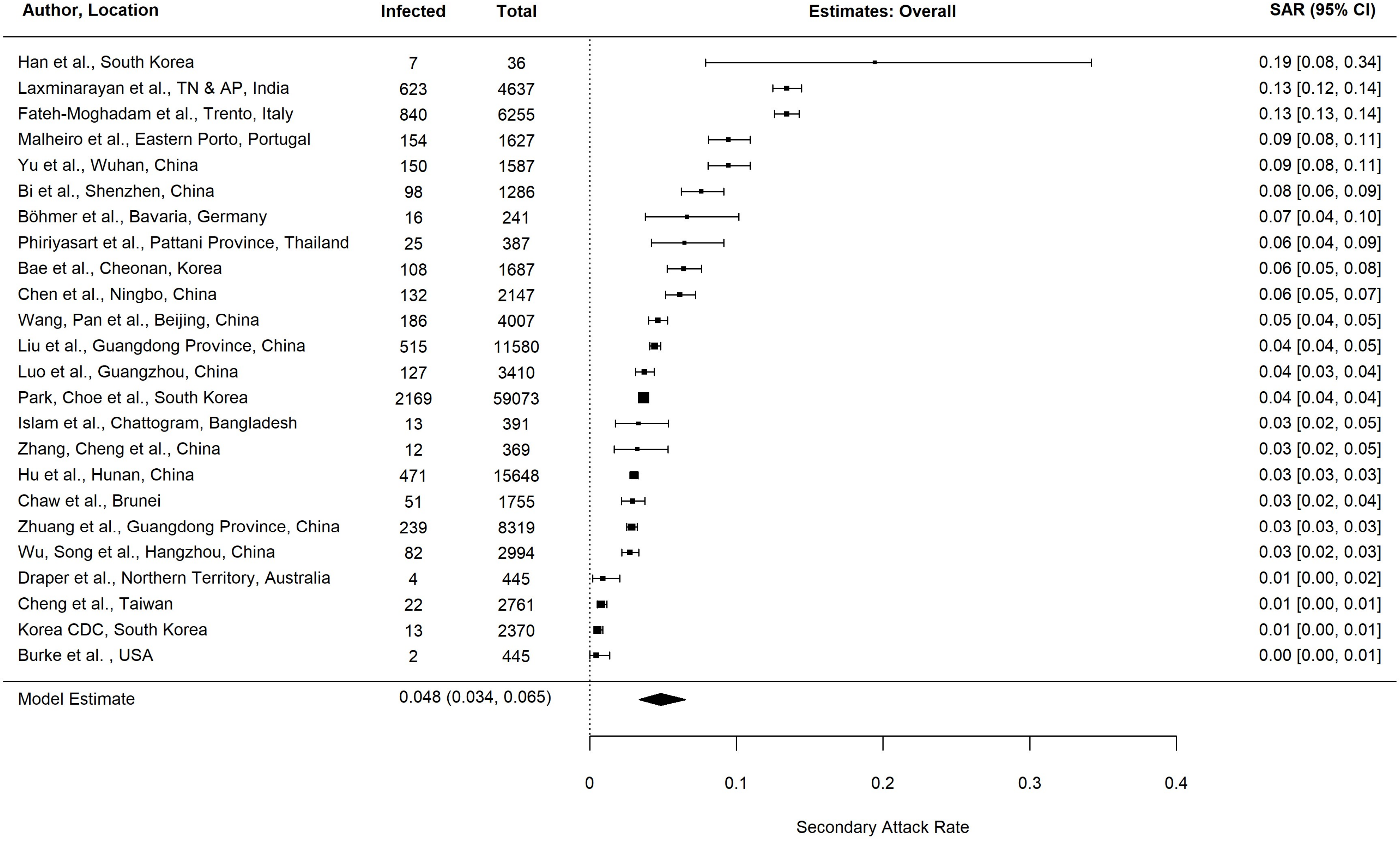

Point sizes are an inverse function of the precision of the estimates and bars correspond to 95% confidence intervals.

© 2020 Madewell ZJ et al. *JAMA Network Open.*

eFigure 3. Funnel Plots of Studies Reporting Secondary Attack Rates of SARS-CoV-2 for Household, Family, and Close Contacts

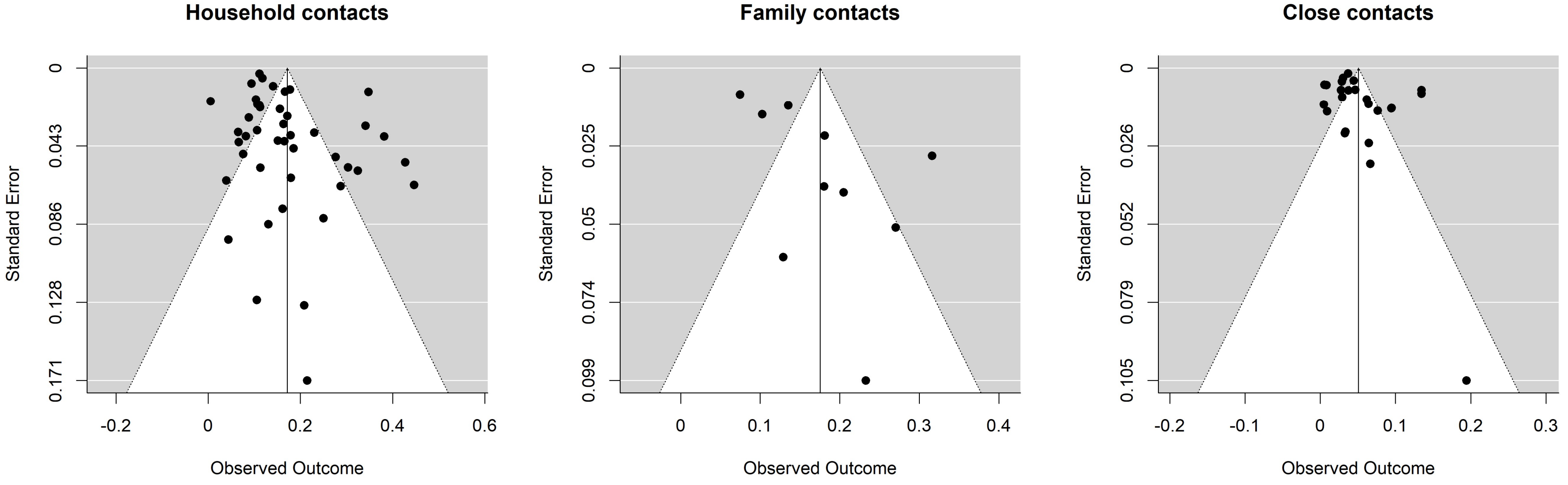

Publication bias assessment: household (Begg: *P*=0.23; Egger: *P*=0.20), family (Begg: *P*=0.48; Egger: *P*=0.14), and close (Begg: *P*=0.16; Egger: *P*=0.17) contacts

© 2020 Madewell ZJ et al. *JAMA Network Open.*

eFigure 4. Household Secondary Attack Rates of SARS-CoV-2, Restricted to Studies With Low or Moderate Risk of Bias as Determined by the Modified Newcastle-Ottawa Scale

See eTable 2 for details

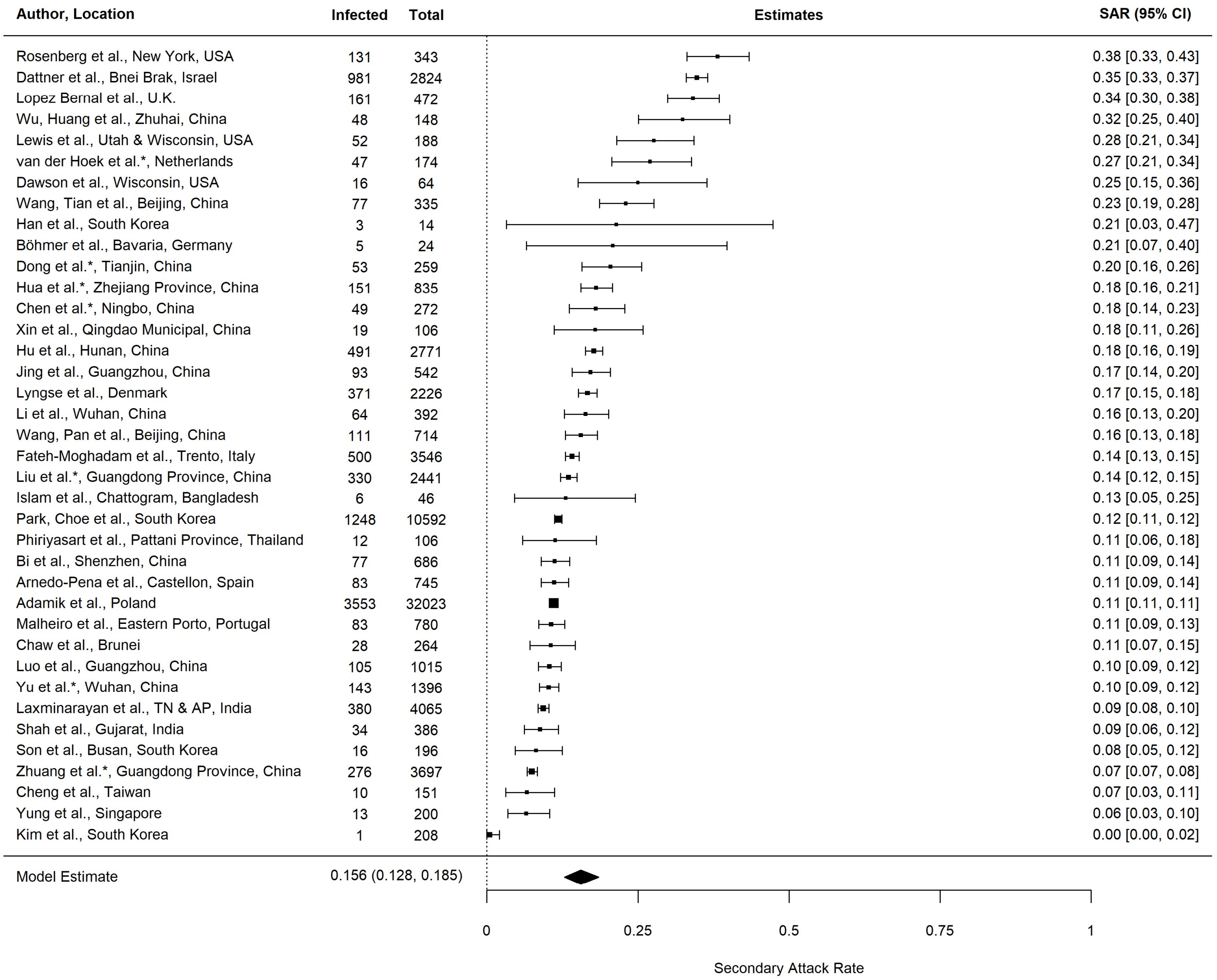

Point sizes are an inverse function of the precision of the estimates and bars correspond to 95% confidence intervals. Studies of family contacts are indicated by the asterisk (*).

© 2020 Madewell ZJ et al. *JAMA Network Open.*

eFigure 5. Household Secondary Attack Rates of SARS-CoV-2, Grouped by Studies in China vs Other Locations

Author, Location	Infected	Total	SAR (95% CI)
**China**			
Wu, Huang et al., Zhuhai	48	148	0.32 [0.25, 0.40]
Sun et al.*, Zhejiang Province	189	598	0.32 [0.28, 0.35]
Wang, Ma et al., Wuhan	47	155	0.30 [0.23, 0.38]
Wang, Zhou et al.*, Wuhan	10	43	0.23 [0.12, 0.37]
Wang, Tian et al., Beijing	77	335	0.23 [0.19, 0.28]
Dong et al.*, Tianjin	53	259	0.20 [0.16, 0.26]
Hua et al.*, Zhejiang Province	151	835	0.18 [0.16, 0.21]
Chen et al.*, Ningbo	49	272	0.18 [0.14, 0.23]
Xin et al., Qingdao Municipal	19	106	0.18 [0.11, 0.26]
Wu, Song et al., Hangzhou	50	280	0.18 [0.14, 0.23]
Hu et al., Hunan	491	2771	0.18 [0.16, 0.19]
Jing et al., Guangzhou	93	542	0.17 [0.14, 0.20]
Li et al., Wuhan	64	392	0.16 [0.13, 0.20]
Zhang, Cheng et al., China	10	62	0.16 [0.08, 0.26]
Wang, Pan et al., Beijing	111	714	0.16 [0.13, 0.18]
Liu et al.*, Guangdong Province	330	2441	0.14 [0.12, 0.15]
Zhang, Zhou et al.*, Liaocheng	12	93	0.13 [0.07, 0.21]
Bi et al., Shenzhen	77	686	0.11 [0.09, 0.14]
Luo et al., Guangzhou	105	1015	0.10 [0.09, 0.12]
Yu et al.*, Wuhan	143	1396	0.10 [0.09, 0.12]
Zhuang et al.*, Guangdong Province	276	3697	0.07 [0.07, 0.08]
Subgroup Estimate	0.174 (0.145, 0.204)		
**Other**			
Boscolo-Rizzo et al., Treviso Province, Italy	54	121	0.45 [0.36, 0.54]
Patel et al., London, UK	79	185	0.43 [0.36, 0.50]
Rosenberg et al., New York, USA	131	343	0.38 [0.33, 0.43]
Dattner et al., Bnei Brak, Israel	981	2824	0.35 [0.33, 0.37]
Lopez Bernal et al., U.K.	161	472	0.34 [0.30, 0.38]
Teherani et al., Atlanta, USA	31	108	0.29 [0.21, 0.38]
Lewis et al., Utah & Wisconsin, USA	52	188	0.28 [0.21, 0.34]
van der Hoek et al.*, Netherlands	47	174	0.27 [0.21, 0.34]
Dawson et al., Wisconsin, USA	16	64	0.25 [0.15, 0.36]
Han et al., South Korea	3	14	0.21 [0.03, 0.47]
Böhmer et al., Bavaria, Germany	5	24	0.21 [0.07, 0.40]
Bae et al., Cheonan, Korea	37	200	0.18 [0.13, 0.24]
Lyngse et al., Denmark	371	2226	0.17 [0.15, 0.18]
Doung-ngern et al., Thailand	38	230	0.17 [0.12, 0.22]
Park, Kim et al., Seoul, South Korea	34	225	0.15 [0.11, 0.20]
Fateh-Moghadam et al., Trento, Italy	500	3546	0.14 [0.13, 0.15]
Islam et al., Chattogram, Bangladesh	6	46	0.13 [0.05, 0.25]
Park, Choe et al., South Korea	1248	10592	0.12 [0.11, 0.12]
Phiriyasart et al., Pattani Province, Thailand	12	106	0.11 [0.06, 0.18]
Arnedo-Pena et al., Castellon, Spain	83	745	0.11 [0.09, 0.14]
Adamik et al., Poland	3553	32023	0.11 [0.11, 0.11]
Malheiro et al., Eastern Porto, Portugal	83	780	0.11 [0.09, 0.13]
Chaw et al., Brunei	28	264	0.11 [0.07, 0.15]
Burke et al., USA	2	19	0.11 [0.00, 0.29]
Laxminarayan et al., TN & AP, India	380	4065	0.09 [0.08, 0.10]
Shah et al., Gujarat, India	34	386	0.09 [0.06, 0.12]
Son et al., Busan, South Korea	16	196	0.08 [0.05, 0.12]
Korea CDC, South Korea	9	119	0.08 [0.03, 0.13]
Cheng et al., Taiwan	10	151	0.07 [0.03, 0.11]
Yung et al., Singapore	13	200	0.06 [0.03, 0.10]
Lee et al., Busan, South Korea	1	23	0.04 [0.00, 0.18]
Draper et al., Northern Territory, Australia	2	51	0.04 [0.00, 0.11]
Kim et al., South Korea	1	208	0.00 [0.00, 0.02]
Subgroup Estimate	0.159 (0.121, 0.201)		
Combined Estimate	0.166 (0.140, 0.193)		

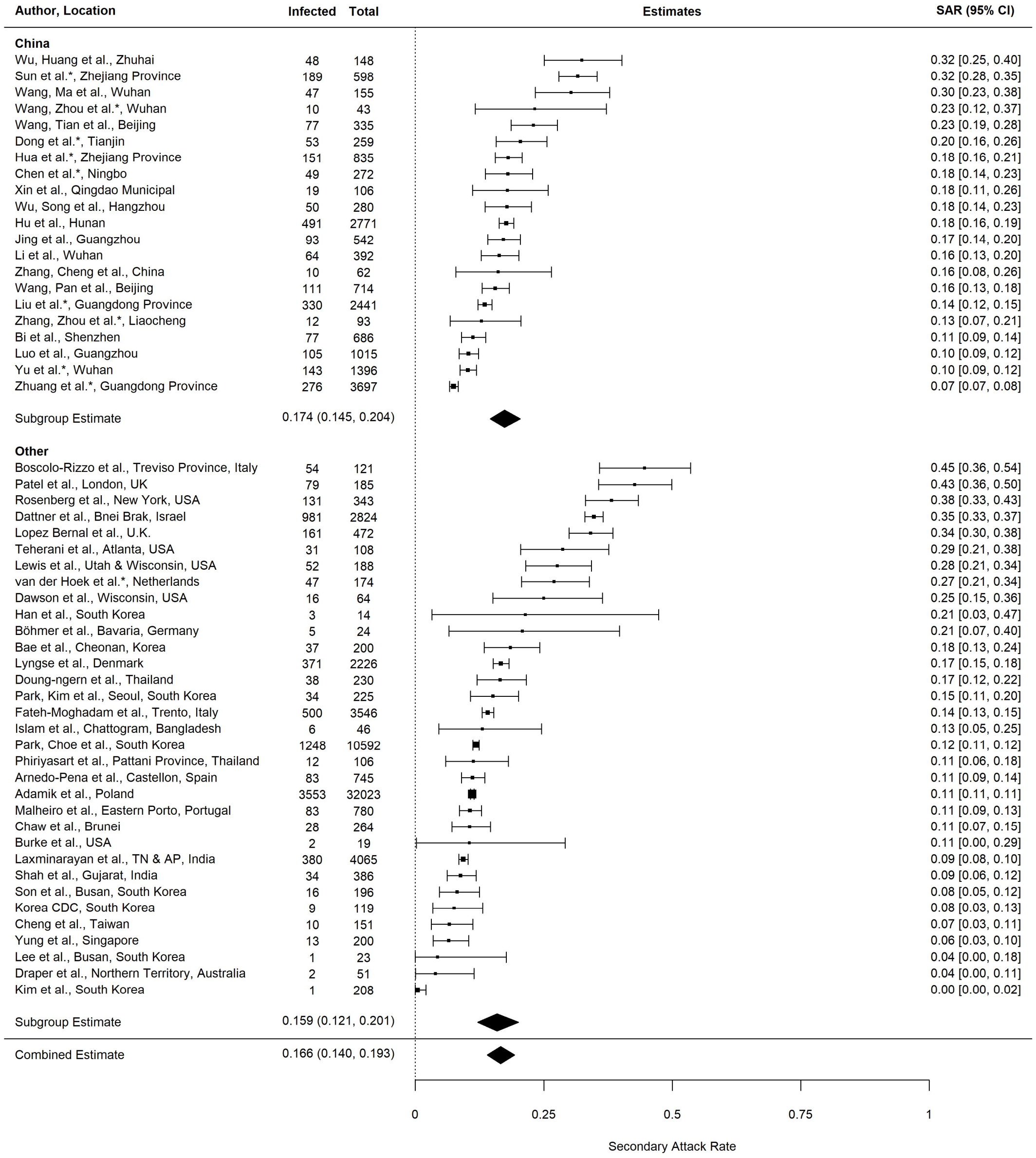

Point sizes are an inverse function of the precision of the estimates and bars correspond to 95% confidence intervals. Studies of family contacts are indicated by the asterisk (*).

© 2020 Madewell ZJ et al. *JAMA Network Open.*

eFigure 6. Secondary Attack Rates of SARS-CoV-2, Grouped by Studies That Tested Only Symptomatic Household Contacts and Studies That Tested All Household Contacts Irrespective of Symptoms

Author, Location	Infected	Total	SAR (95% CI)
**Tested symptomatic**			
Boscolo-Rizzo et al., Treviso Province, Italy	54	121	0.45 [0.36, 0.54]
Patel et al., London, UK	79	185	0.43 [0.36, 0.50]
Dattner et al., Bnei Brak, Israel	981	2824	0.35 [0.33, 0.37]
Lopez Bernal et al., U.K.	161	472	0.34 [0.30, 0.38]
Sun et al.*, Zhejiang Province, China	189	598	0.32 [0.28, 0.35]
Wang, Ma et al., Wuhan, China	47	155	0.30 [0.23, 0.38]
Wang, Zhou et al.*, Wuhan, China	10	43	0.23 [0.12, 0.37]
Teherani et al., Atlanta, USA	31	144	0.22 [0.15, 0.29]
Bae et al., Cheonan, Korea	37	200	0.18 [0.13, 0.24]
Fateh-Moghadam et al., Trento, Italy	500	3546	0.14 [0.13, 0.15]
Arnedo-Pena et al., Castellon, Spain	83	745	0.11 [0.09, 0.14]
Adamik et al., Poland	3553	32023	0.11 [0.11, 0.11]
Malheiro et al., Eastern Porto, Portugal	83	780	0.11 [0.09, 0.13]
Burke et al., USA	2	19	0.11 [0.00, 0.29]
Shah et al., Gujarat, India	34	386	0.09 [0.06, 0.12]
Korea CDC, South Korea	9	119	0.08 [0.03, 0.13]
Cheng et al., Taiwan	10	151	0.07 [0.03, 0.11]
Draper et al., Northern Territory, Australia	2	51	0.04 [0.00, 0.11]
Subgroup Estimate	0.191 (0.135, 0.254)		
**Tested symptomatic and asymptomatic**			
Rosenberg et al., New York, USA	131	343	0.38 [0.33, 0.43]
Wu, Huang et al., Zhuhai, China	48	148	0.32 [0.25, 0.40]
Lewis et al., Utah & Wisconsin, USA	52	188	0.28 [0.21, 0.34]
van der Hoek et al.*, Netherlands	47	174	0.27 [0.21, 0.34]
Dawson et al., Wisconsin, USA	16	64	0.25 [0.15, 0.36]
Wang, Tian et al., Beijing, China	77	335	0.23 [0.19, 0.28]
Han et al., South Korea	3	14	0.21 [0.03, 0.47]
Böhmer et al., Bavaria, Germany	5	24	0.21 [0.07, 0.40]
Dong et al.*, Tianjin, China	53	259	0.20 [0.16, 0.26]
Hua et al.*, Zhejiang Province, China	151	835	0.18 [0.16, 0.21]
Chen et al.*, Ningbo, China	49	272	0.18 [0.14, 0.23]
Xin et al., Qingdao Municipal, China	19	106	0.18 [0.11, 0.26]
Wu, Song et al., Hangzhou, China	50	280	0.18 [0.14, 0.23]
Hu et al., Hunan, China	491	2771	0.18 [0.16, 0.19]
Jing et al., Guangzhou, China	93	542	0.17 [0.14, 0.20]
Lyngse et al., Denmark	371	2226	0.17 [0.15, 0.18]
Li et al., Wuhan, China	64	392	0.16 [0.13, 0.20]
Zhang, Cheng et al., China	10	62	0.16 [0.08, 0.26]
Park, Kim et al., Seoul, South Korea	34	225	0.15 [0.11, 0.20]
Liu et al.*, Guangdong Province, China	330	2441	0.14 [0.12, 0.15]
Islam et al., Chattogram, Bangladesh	6	46	0.13 [0.05, 0.25]
Zhang, Zhou et al.*, Liaocheng, China	12	93	0.13 [0.07, 0.21]
Park, Choe et al., South Korea	1248	10592	0.12 [0.11, 0.12]
Phiriyasart et al., Pattani Province, Thailand	12	106	0.11 [0.06, 0.18]
Bi et al., Shenzhen, China	77	686	0.11 [0.09, 0.14]
Chaw et al., Brunei	28	264	0.11 [0.07, 0.15]
Luo et al., Guangzhou, China	105	1015	0.10 [0.09, 0.12]
Laxminarayan et al., TN & AP, India	380	4065	0.09 [0.08, 0.10]
Son et al., Busan, South Korea	16	196	0.08 [0.05, 0.12]
Zhuang et al.*, Guangdong Province, China	276	3697	0.07 [0.07, 0.08]
Yung et al., Singapore	13	200	0.06 [0.03, 0.10]
Lee et al., Busan, South Korea	1	23	0.04 [0.00, 0.18]
Kim et al., South Korea	1	208	0.00 [0.00, 0.02]
Subgroup Estimate	0.153 (0.125, 0.182)		

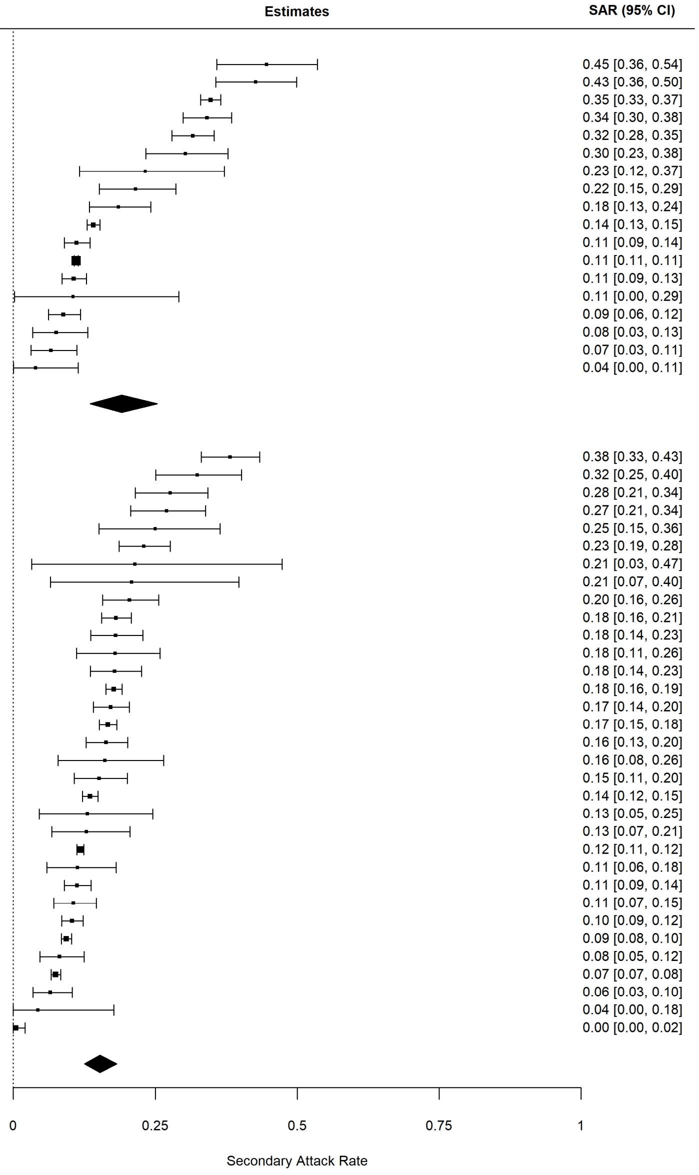

Three studies were excluded from this analysis: two for which we could not determine whether they tested only symptomatic or all contacts, and one that tested only asymptomatic contacts. Point sizes are an inverse function of the precision of the estimates and bars correspond to 95% confidence intervals. Studies of family contacts are indicated by the asterisk (*).

© 2020 Madewell ZJ et al. *JAMA Network Open.*

eFigure 7. Household Secondary Attack Rates of SARS-CoV-2, Grouped by Studies Early (January-February) and Later (March-July) in the Pandemic

Author, Location	Infected	Total	SAR (95% CI)
**January-February**			
Wu, Huang et al., Zhuhai, China	48	148	0.32 [0.25, 0.40]
Sun et al.*, Zhejiang Province, China	189	598	0.32 [0.28, 0.35]
Wang, Ma et al., Wuhan, China	47	155	0.30 [0.23, 0.38]
Wang, Zhou et al.*, Wuhan, China	10	43	0.23 [0.12, 0.37]
Wang, Tian et al., Beijing, China	77	335	0.23 [0.19, 0.28]
Böhmer et al., Bavaria, Germany	5	24	0.21 [0.07, 0.40]
Dong et al.*, Tianjin, China	53	259	0.20 [0.16, 0.26]
Hua et al.*, Zhejiang Province, China	151	835	0.18 [0.16, 0.21]
Wu, Song et al., Hangzhou, China	50	280	0.18 [0.14, 0.23]
Jing et al., Guangzhou, China	93	542	0.17 [0.14, 0.20]
Li et al., Wuhan, China	64	392	0.16 [0.13, 0.20]
Zhang, Zhou et al.*, Liaocheng, China	12	93	0.13 [0.07, 0.21]
Bi et al., Shenzhen, China	77	686	0.11 [0.09, 0.14]
Burke et al., USA	2	19	0.11 [0.00, 0.29]
Yu et al.*, Wuhan, China	143	1396	0.10 [0.09, 0.12]
Zhuang et al.*, Guangdong Province, China	276	3697	0.07 [0.07, 0.08]
Subgroup Estimate	0.189 (0.149, 0.232)		
**March-July**			
Boscolo-Rizzo et al., Treviso Province, Italy	54	121	0.45 [0.36, 0.54]
Patel et al., London, UK	79	185	0.43 [0.36, 0.50]
Rosenberg et al., New York, USA	131	343	0.38 [0.33, 0.43]
Dattner et al., Bnei Brak, Israel	981	2824	0.35 [0.33, 0.37]
Teherani et al., Atlanta, USA	31	108	0.29 [0.21, 0.38]
Lewis et al., Utah & Wisconsin, USA	52	188	0.28 [0.21, 0.34]
van der Hoek et al.*, Netherlands	47	174	0.27 [0.21, 0.34]
Dawson et al., Wisconsin, USA	16	64	0.25 [0.15, 0.36]
Han et al., South Korea	3	14	0.21 [0.03, 0.47]
Doung-ngern et al., Thailand	38	230	0.17 [0.12, 0.22]
Fateh-Moghadam et al., Trento, Italy	500	3546	0.14 [0.13, 0.15]
Islam et al., Chattogram, Bangladesh	6	46	0.13 [0.05, 0.25]
Phiriyasart et al., Pattani Province, Thailand	12	106	0.11 [0.06, 0.18]
Adamik et al., Poland	3553	32023	0.11 [0.11, 0.11]
Malheiro et al., Eastern Porto, Portugal	83	780	0.11 [0.09, 0.13]
Chaw et al., Brunei	28	264	0.11 [0.07, 0.15]
Laxminarayan et al., TN & AP, India	380	4065	0.09 [0.08, 0.10]
Shah et al., Gujarat, India	34	386	0.09 [0.06, 0.12]
Yung et al., Singapore	13	200	0.06 [0.03, 0.10]
Draper et al., Northern Territory, Australia	2	51	0.04 [0.00, 0.11]
Subgroup Estimate	0.179 (0.121, 0.245)		

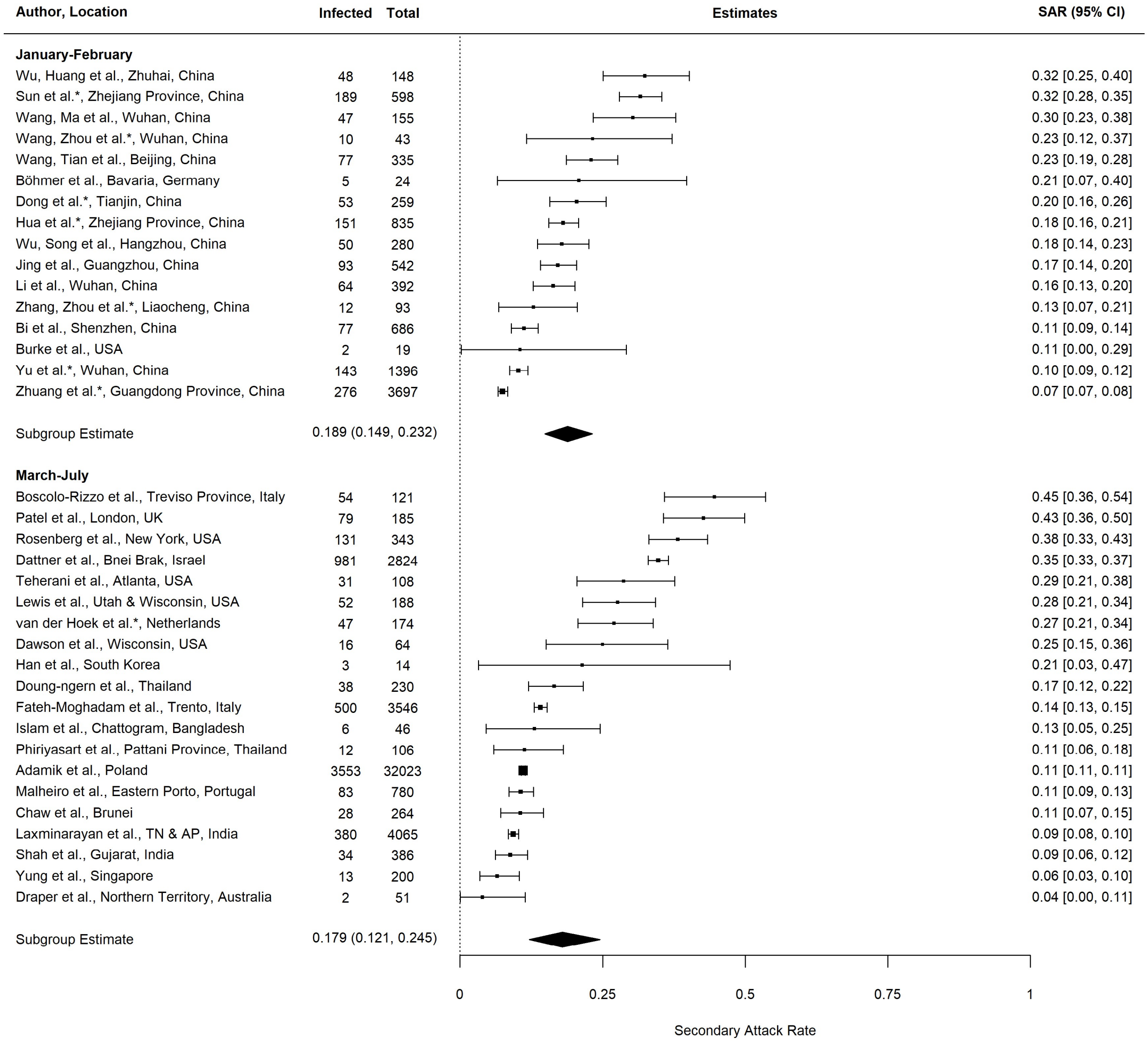

This analysis excluded studies with overlapping dates (e.g., studies that began in February and ended in April). Point sizes are an inverse function of the precision of the estimates and bars correspond to 95% confidence intervals. Studies of family contacts are indicated by the asterisk (*).

© 2020 Madewell ZJ et al. *JAMA Network Open.*

eFigure 8. Secondary Attack Rates of SARS-CoV-2 From Symptomatic and Asymptomatic or Presymptomatic Index Cases to Household and Family Contacts

Author, Location	Infected	Total	SAR (95% CI)
**Symptomatic**			
Boscolo-Rizzo et al., Treviso Province, Italy	54	121	0.45 [0.36, 0.54]
Patel et al., London, UK	79	185	0.43 [0.36, 0.50]
Lopez Bernal et al., U.K.	161	472	0.34 [0.30, 0.38]
Wang, Ma et al., Wuhan, China	47	155	0.30 [0.23, 0.38]
Teherani et al., Atlanta, USA	31	108	0.29 [0.21, 0.38]
Lewis et al., Utah & Wisconsin, USA	52	186	0.28 [0.22, 0.35]
van der Hoek et al.*, Netherlands	47	174	0.27 [0.21, 0.34]
Dawson et al., Wisconsin, USA	16	64	0.25 [0.15, 0.36]
Wang, Zhou et al.*, Wuhan, China	10	43	0.23 [0.12, 0.37]
Wang, Tian et al., Beijing, China	77	335	0.23 [0.19, 0.28]
Böhmer et al., Bavaria, Germany	5	24	0.21 [0.07, 0.40]
Bae et al., Cheonan, Korea	37	200	0.18 [0.13, 0.24]
Doung-ngern et al., Thailand	38	230	0.17 [0.12, 0.22]
Li et al., Wuhan, China	64	392	0.16 [0.13, 0.20]
Park, Kim et al., Seoul, South Korea	34	210	0.16 [0.11, 0.22]
Chaw et al., Brunei	22	153	0.14 [0.09, 0.20]
Fateh-Moghadam et al., Trento, Italy	500	3546	0.14 [0.13, 0.15]
Phiriyasart et al., Pattani Province, Thailand	12	106	0.11 [0.06, 0.18]
Arnedo-Pena et al., Castellon, Spain	83	745	0.11 [0.09, 0.14]
Adamik et al., Poland	3553	32023	0.11 [0.11, 0.11]
Malheiro et al., Eastern Porto, Portugal	83	780	0.11 [0.09, 0.13]
Burke et al., USA	2	19	0.11 [0.00, 0.29]
Yu et al.*, Wuhan, China	143	1396	0.10 [0.09, 0.12]
Shah et al., Gujarat, India	34	386	0.09 [0.06, 0.12]
Zhuang et al.*, Guangdong Province, China	276	3697	0.07 [0.07, 0.08]
Yung et al., Singapore	13	200	0.06 [0.03, 0.10]
Draper et al., Northern Territory, Australia	2	51	0.04 [0.00, 0.11]
Subgroup Estimate	0.180 (0.142, 0.221)		
**Asymptomatic or Presymptomatic**			
Chaw et al., Brunei	6	111	0.05 [0.02, 0.11]
Lee et al., Busan, South Korea	1	23	0.04 [0.00, 0.18]
Lewis et al., Utah & Wisconsin, USA	0	2	0.00 [0.00, 0.70]
Park, Kim et al., Seoul, South Korea	0	15	0.00 [0.00, 0.11]
Subgroup Estimate	0.007 (0.000, 0.049)		

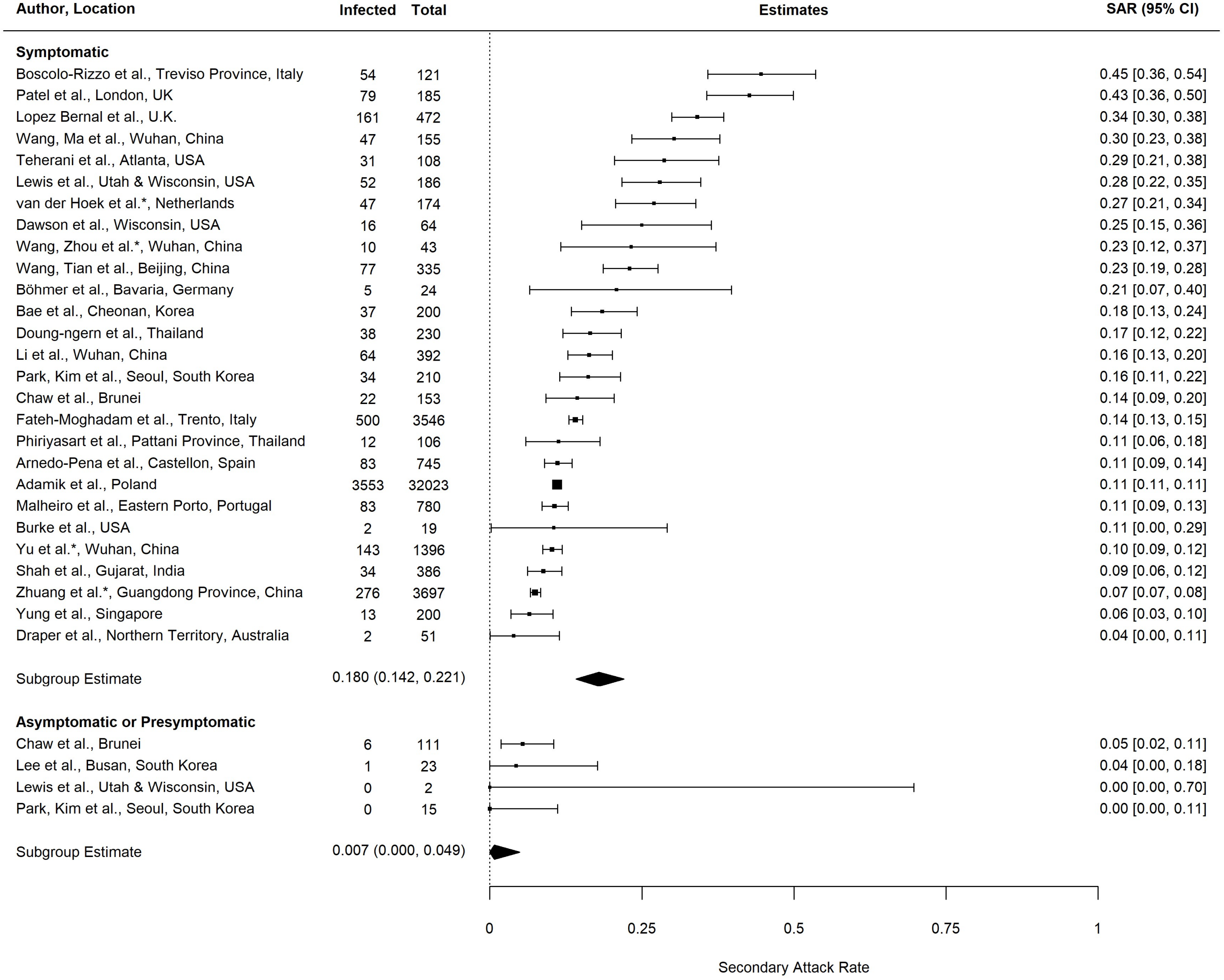

Studies of family contacts are indicated by the asterisk (*). Point sizes are an inverse function of the precision of the estimates and bars correspond to 95% confidence intervals.

© 2020 Madewell ZJ et al. *JAMA Network Open.*

eFigure 9. Funnel Plots of Studies Reporting Household Secondary Attack Rates of SARS-CoV-2 for Adult (≥18 Years) and Child (<18 Years) Contacts

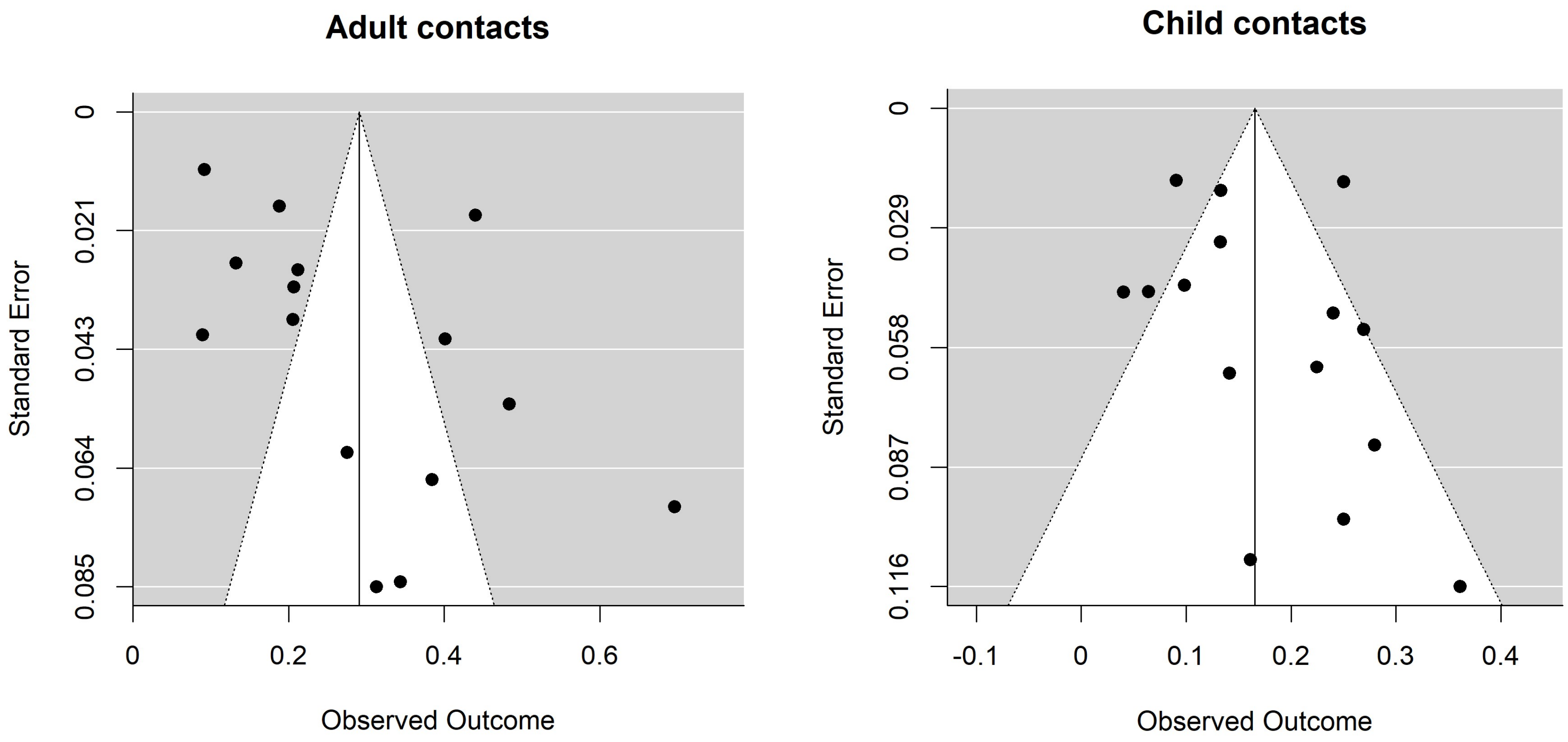

Publication bias assessment: Adult (Begg: *P*=0.03; Egger: *P*=0.03) and child (Begg: *P*=0.20; Egger: *P*=0.12) contacts

© 2020 Madewell ZJ et al. *JAMA Network Open.*

eFigure 10. Secondary Attack Rates of SARS-CoV-2 for Household and Family Contacts by Contact Sex

Author, Location	Infected	Total	SAR (95% CI)
***Lopez Bernal et al.*, U.K.**			
Female	80	241	0.33 [0.27, 0.39]
Male	81	231	0.35 [0.29, 0.41]
***Wu, Huang et al.*, Zhuhai, China**			
Female	29	80	0.36 [0.26, 0.47]
Male	19	63	0.30 [0.19, 0.42]
***Sun et al.**, Zhejiang Province, China**			
Female	101	320	0.32 [0.27, 0.37]
Male	88	278	0.32 [0.26, 0.37]
***Lewis et al.*, Utah & Wisconsin, USA**			
Female	31	96	0.32 [0.23, 0.42]
Male	21	92	0.23 [0.15, 0.32]
***Xin et al.*, Qingdao Municipal, China**			
Female	11	51	0.22 [0.11, 0.34]
Male	8	55	0.15 [0.06, 0.25]
***Jing et al.*, Guangzhou, China**			
Female	53	280	0.19 [0.15, 0.24]
Male	40	258	0.16 [0.11, 0.20]
***Lyngse et al.*, Denmark**			
Female	198	1103	0.18 [0.16, 0.20]
Male	173	1123	0.15 [0.13, 0.18]
***Li et al.*, Wuhan, China**			
Female	31	181	0.17 [0.12, 0.23]
Male	33	211	0.16 [0.11, 0.21]
***Arnedo-Pena et al.*, Castellon, Spain**			
Female	45	409	0.11 [0.08, 0.14]
Male	38	336	0.11 [0.08, 0.15]
***Laxminarayan et al.*, TN & AP, India**			
Female	201	1754	0.11 [0.10, 0.13]
Male	147	1625	0.09 [0.08, 0.10]
***Yung et al.*, Singapore**			
Female	8	104	0.08 [0.03, 0.14]
Male	5	96	0.05 [0.01, 0.11]
Female Estimate	0.207 (0.150, 0.269)		
Male Estimate	0.177 (0.124, 0.238)		

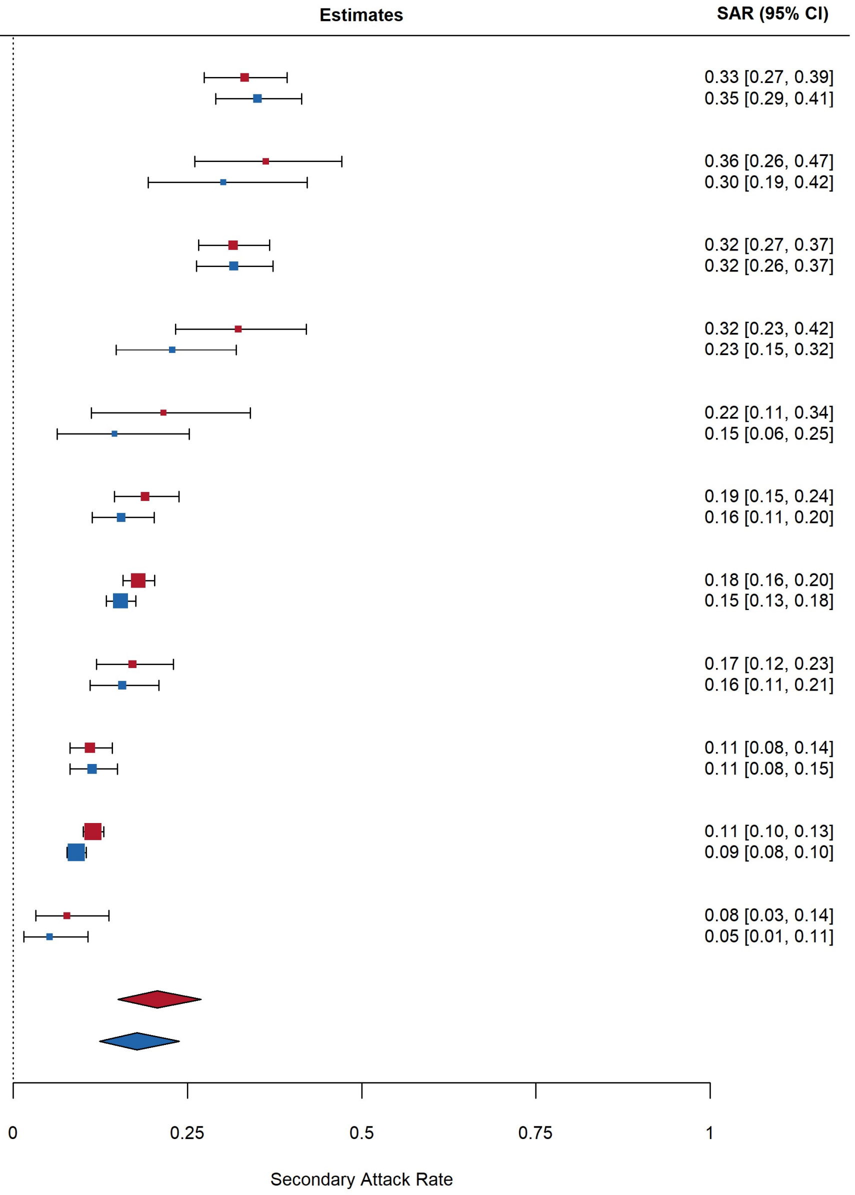

Point sizes are an inverse function of the precision of the estimates and bars correspond to 95% confidence intervals. Studies of family contacts are indicated by the asterisk (*). Red: female; blue: male.

© 2020 Madewell ZJ et al. *JAMA Network Open.*

eFigure 11. Funnel Plots of Studies Reporting Household Secondary Attack Rates of SARS-CoV-2 for Female and Male Contacts

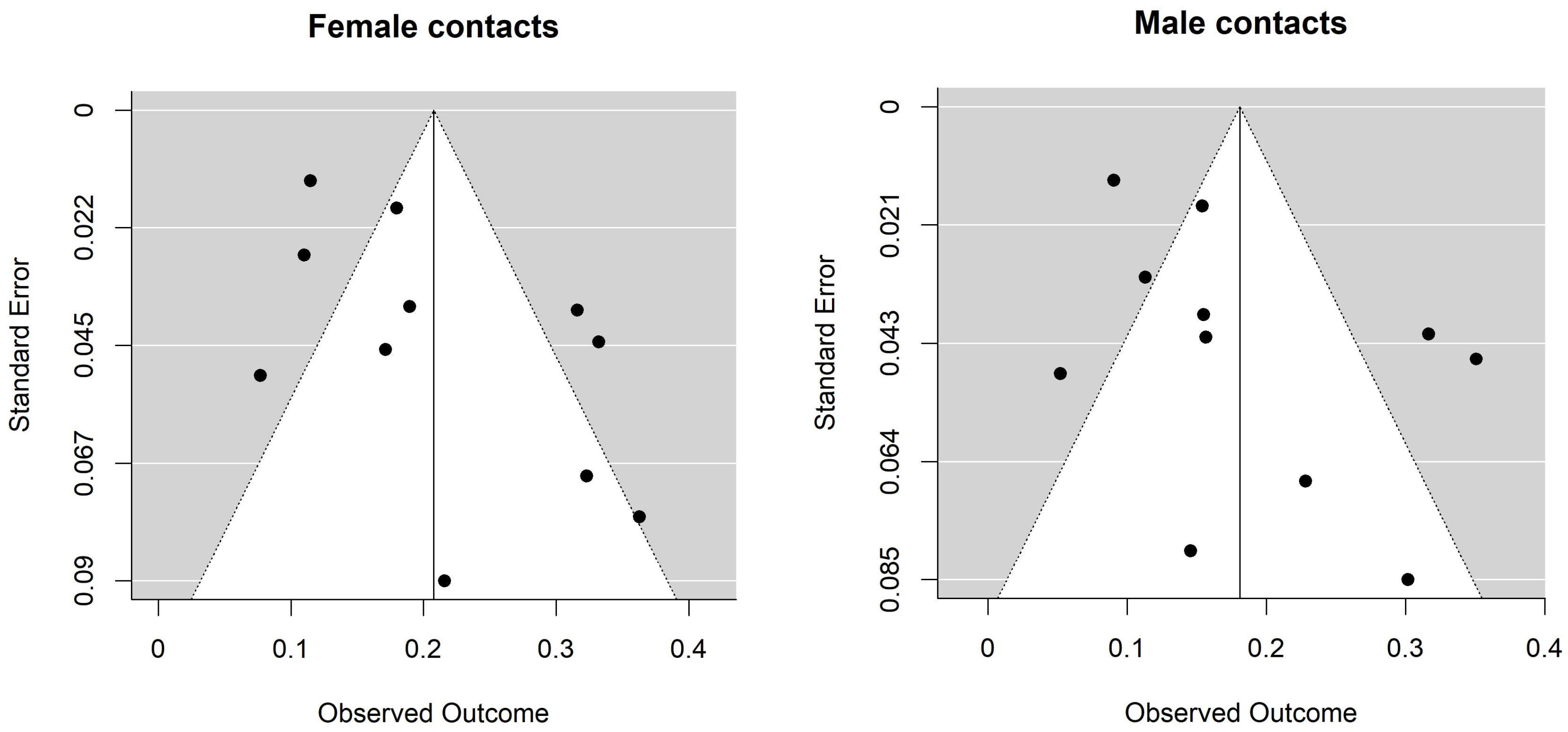

Publication bias assessment: female (Begg: *P*=0.45; Egger: *P*=0.07) and male contacts (Begg: *P*=0.36; Egger: *P*=0.20)

© 2020 Madewell ZJ et al. *JAMA Network Open.*

eFigure 12. Secondary Attack Rates of SARS-CoV-2 to Household Contacts From Adult (≥18 Years) and Child (<18 Years) Index Cases

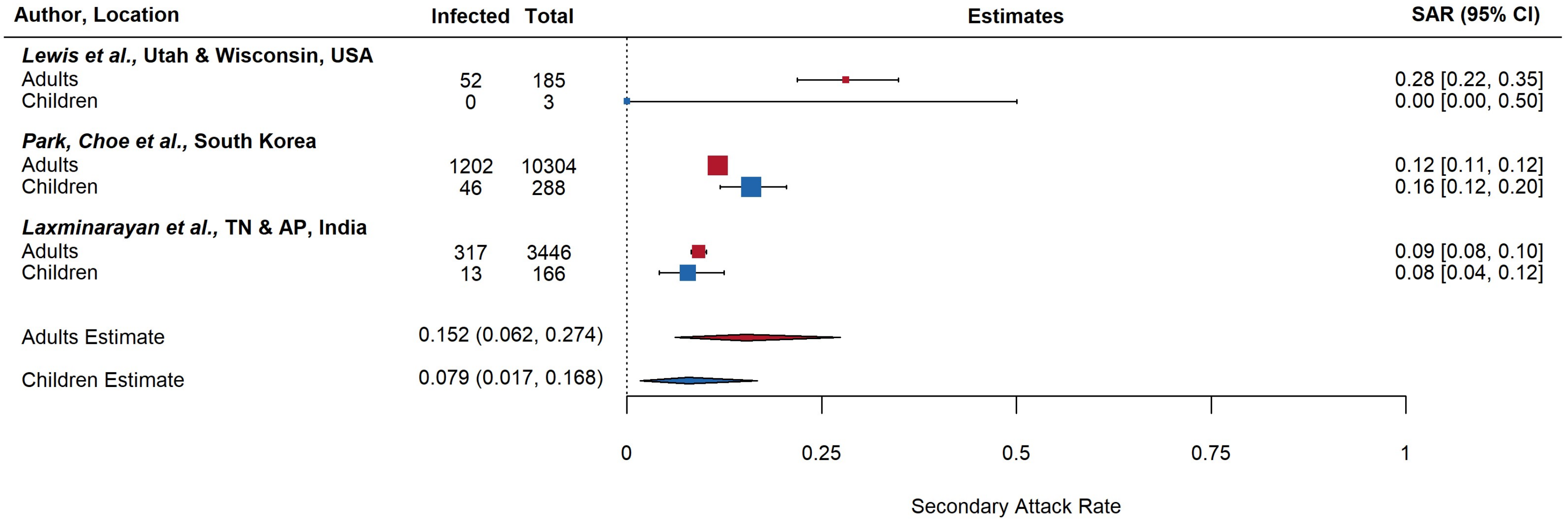

Point sizes are an inverse function of the precision of the estimates and bars correspond to 95% confidence intervals. Red: adults; blue: children.

© 2020 Madewell ZJ et al. *JAMA Network Open.*

eFigure 13. Secondary Attack Rates of SARS-CoV-2 for Household Contacts by Index Case Sex

Author, Location	Infected	Total	SAR (95% CI)
***Wu, Huang et al.*, Zhuhai, China**			
Female	21	66	0.32 [0.21, 0.44]
Male	27	77	0.35 [0.25, 0.46]
***Lewis et al.*, Utah & Wisconsin, USA**			
Female	15	84	0.18 [0.10, 0.27]
Male	37	104	0.36 [0.27, 0.45]
***Xin et al.*, Qingdao Municipal, China**			
Female	14	51	0.27 [0.16, 0.41]
Male	5	55	0.09 [0.03, 0.18]
***Li et al.*, Wuhan, China**			
Female	29	183	0.16 [0.11, 0.22]
Male	35	209	0.17 [0.12, 0.22]
***Arnedo-Pena et al.*, Castellon, Spain**			
Female	47	401	0.12 [0.09, 0.15]
Male	36	344	0.10 [0.07, 0.14]
***Laxminarayan et al.*, TN & AP, India**			
Female	85	883	0.10 [0.08, 0.12]
Male	263	2496	0.11 [0.09, 0.12]
***Yung et al.*, Singapore**			
Female	7	56	0.12 [0.05, 0.23]
Male	7	97	0.07 [0.03, 0.13]
Female Estimate	0.166 (0.112, 0.228)		
Male Estimate	0.164 (0.090, 0.255)		

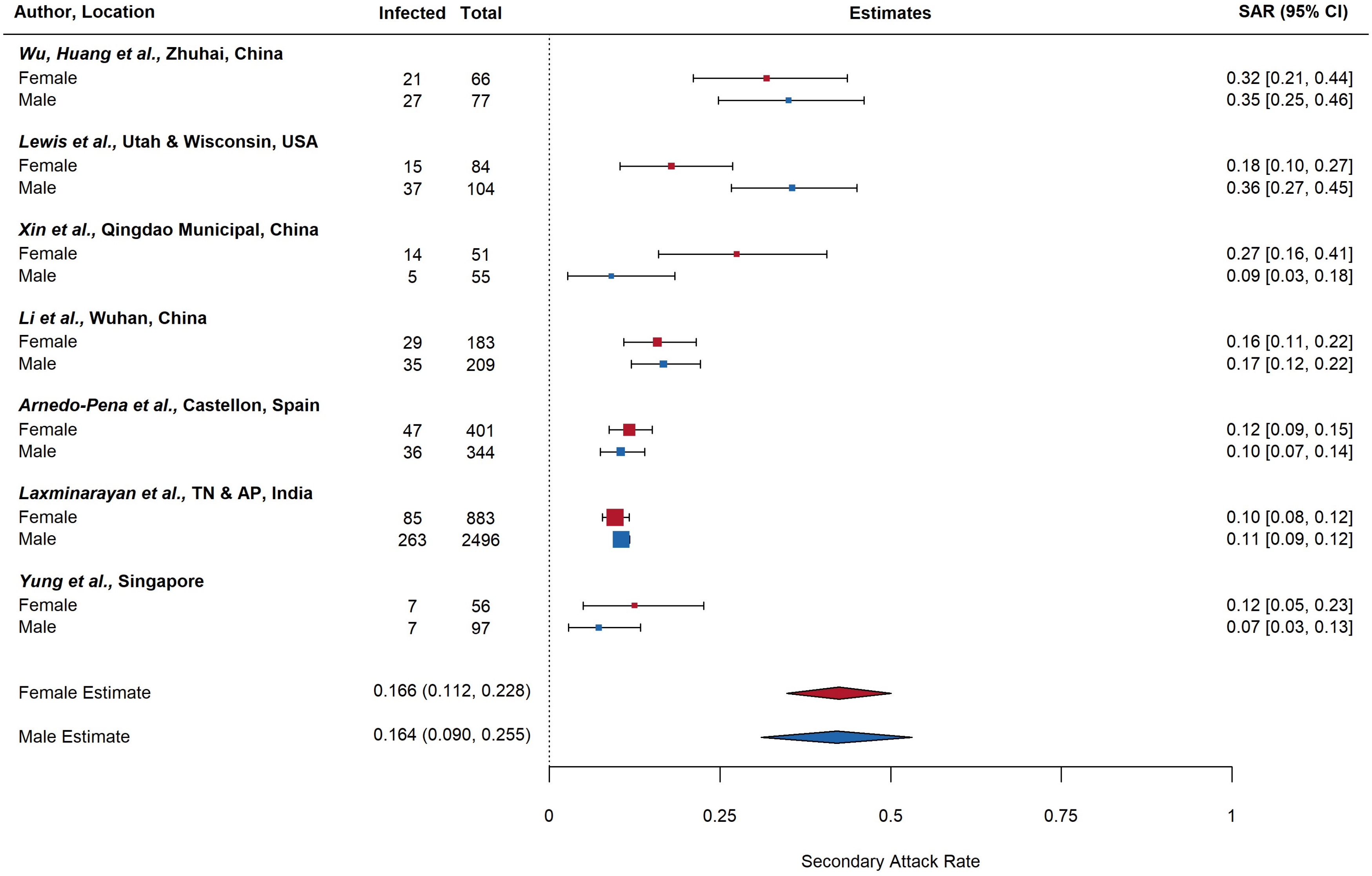

Point sizes are an inverse function of the precision of the estimates and bars correspond to 95% confidence intervals. Red: female; blue: male.

© 2020 Madewell ZJ et al. *JAMA Network Open.*

eFigure 14. Household Secondary Attack Rates of SARS-CoV and MERS-CoV

Author, Location	Infected	Total	SAR (95% CI)
**SARS-CoV**			
Chan et al., Hong Kong, China	30	243	0.12 [0.08, 0.17]
Pang et al., Beijing, China	111	973	0.11 [0.09, 0.13]
Wilson-Clark et al., Toronto, Canada	18	176	0.10 [0.06, 0.15]
Lau et al., Hong Kong, China	188	2139	0.09 [0.08, 0.10]
Goh et al., Singapore	26	417	0.06 [0.04, 0.09]
Ou et al., Beijing, China	18	550	0.03 [0.02, 0.05]
Tuan et al., Vietnam	3	123	0.02 [0.00, 0.06]
Subgroup Estimate	0.075 (0.048, 0.107)		
**MERS-CoV**			
Arwady et al.*, Al-Qouz, Saudi Arabia	19	79	0.24 [0.15, 0.34]
Payne et al., Jordan	2	23	0.09 [0.00, 0.24]
Van Kerkhove et al., Riyadh, Saudi Arabia	12	235	0.05 [0.03, 0.08]
Drosten et al., Saudi Arabia	12	280	0.04 [0.02, 0.07]
Assiri et al., Al-Hasa, Saudi Arabia	5	217	0.02 [0.01, 0.05]
Memish et al., Al-Madinah al-Munawwarah, Saudi Arabia	1	56	0.02 [0.00, 0.08]
Al Hosani et al., Abu Dhabi, United Arab Emirates	0	105	0.00 [0.00, 0.02]
Subgroup Estimate	0.047 (0.009, 0.107)		

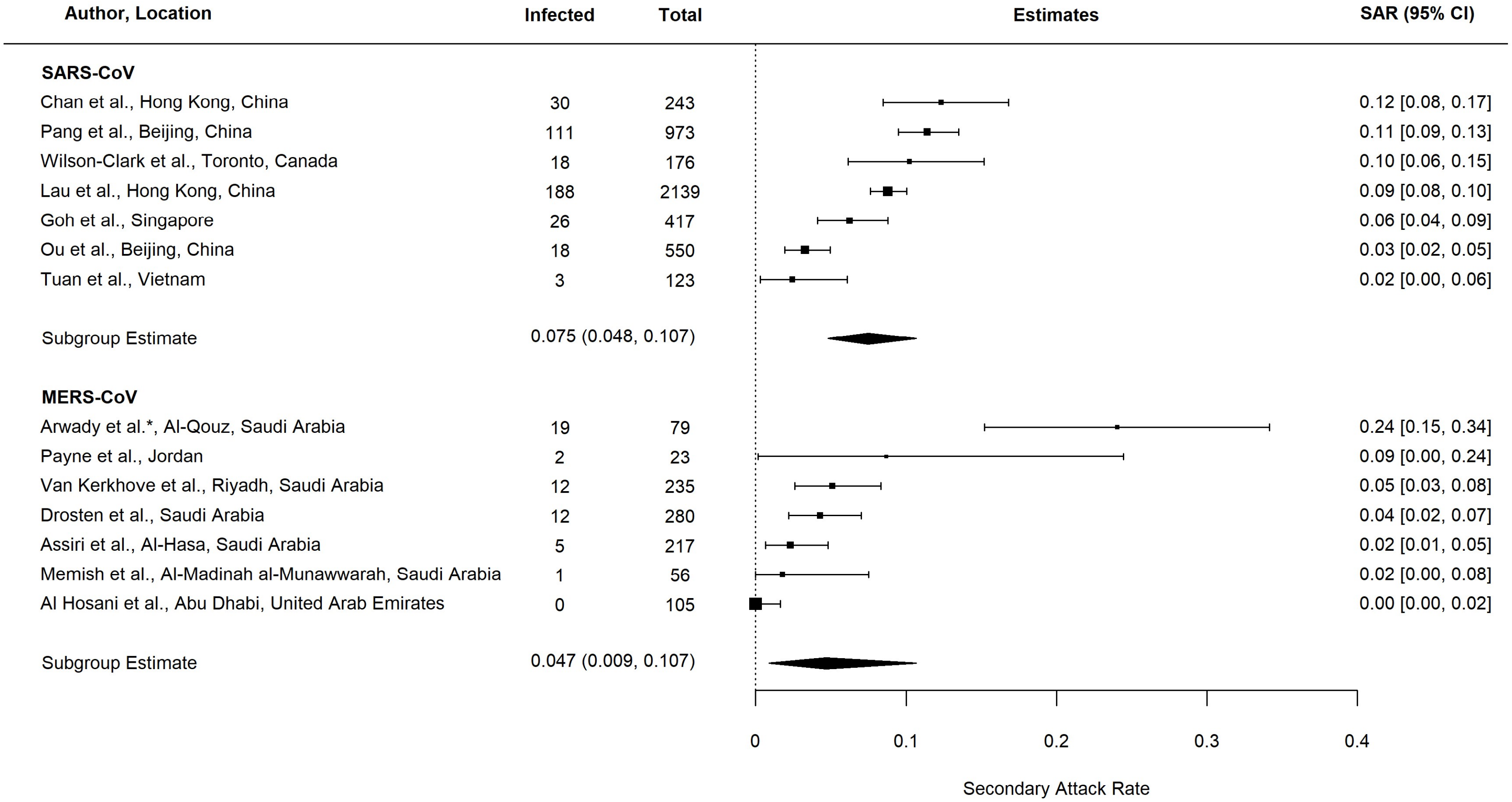

Point sizes are an inverse function of the precision of the estimates and bars correspond to 95% confidence intervals. Studies of family contacts are indicated by the asterisk (*).

© 2020 Madewell ZJ et al. *JAMA Network Open.*

eTable 1. Electronic Databases and Search Strategy for Household Secondary Attack Rate of SARS-CoV-2, MERS-CoV, SARS-CoV, and Other Coronaviruses

**Database: PubMed: 1,252 retrieved articles**	
#1: "SARS-CoV-2" [All Fields] OR COVID-19 [All Fields] OR "MERS" [All Fields] OR "MERS-CoV" [All Fields] OR "Middle East Respiratory Syndrome" [All Fields] OR "SARS-CoV" [All Fields] OR "SARS" [All Fields] OR "severe acute respiratory syndrome" [All Fields] OR "coronavirus" [All Fields] OR "HCoV-NL63" [All Fields] OR "HCoV-OC43" [All Fields] OR "HCoV-229E" [All Fields] OR "HCoV-HKU1" [All Fields] OR "HCoV" [All Fields] OR "Human coronavirus NL63" [All Fields] OR "Human coronavirus OC43" [All Fields] OR "Human coronavirus 229E" [All Fields] OR "Human coronavirus HKU1" [All Fields])	#2: "secondary attack rate" [All Fields] OR household [All Fields] OR "close contacts" [All Fields] OR "contact transmission" [All Fields] OR "contact attack rate" [All Fields] OR "family transmission" [All Fields]
**#1 AND #2**	

We searched PubMed using terms described in this table for studies of SARS-CoV-2, SARS-CoV, Middle East Respiratory Syndrome coronavirus (MERS-CoV), and human coronaviruses NL63 (HCoV-NL63), OC43 (HCoV-OC43), 229E (HCoV-229E), and HKU1 (HCoV-HKU1) with no restrictions on language, study design, time, or place of publication. Pre-prints were included. We also manually searched the reference lists of eligible studies from PubMed to identify further eligible studies. For influenza, we used the secondary attack rates reported in one comprehensive review.[1] We managed all citations using Endnote software X8 (Thomson Reuters, Toronto, CA, USA).

© 2020 Madewell ZJ et al. *JAMA Network Open.*

eTable 2. Risk of Bias Assessment for Studies Included in Review of Household Transmissibility of SARS-CoV-2

	Selection			Comparability	Outcome				
**Author**	**Representativeness of the index cases in region (2 points)[a]**	**Index case definition (1 point)[b]**	**Sample size (1 point)[c]**	**Household secondary attack rate disaggregated by index and/or contact covariates (1 point)[d]**	**Universal or symptom-based testing (1 point)[e]**	**Follow-up duration (2 points)[f]**	**Number of tests per contact (1 point)[g]**	**Total points**	**Risk of bias[h]**
Adamik et al.[4]	++	+	+	+	0	0	0	5	Moderate
Arnedo-Pena et al.[5]	++	0	+	+	0	0	0	4	Moderate
Bae et al.[6]	0	+	0	0	0	+	0	2	High
Bi et al.[7]	++	+	+	+	+	+	0	7	Low
Böhmer et al.[8]	0	+	0	+	+	+	+	5	Moderate
Boscolo-Rizzo et al.[9]	+	0	0	0	0	+	0	2	High
Burke et al.[10]	0	+	0	0	0	+	0	2	High
Chaw et al.[11]	++	+	0	+	+	+	+	7	Low
Chen et al.[12]	++	+	0	+	+	++	+	8	Low
Cheng et al.[13]	++	+	0	0	0	+	+	5	Moderate
Dattner et al.[14]	++	+	+	+	0	0	0	5	Moderate
Dawson et al.[15]	+	+	0	0	+	0	+	4	Moderate
Dong et al.[16]	++	+	0	0	+	0	+	5	Moderate
Doung-ngern et al.[17]	0	+	0	0	0	++	0	3	High
Draper et al.[18]	++	0	0	0	0	+	0	3	High
Fateh-Moghadam et al.[19]	+	+	+	0	0	+	0	4	Moderate
Han et al.[20]	0	+	0	0	+	+	+	4	Moderate
Hu et al.[21]	++	+	+	0	+	+	0	6	Moderate
Hua et al.[22]	++	+	+	+	+	0	0	6	Moderate
Islam et al.[23]	+	+	0	0	+	+	0	4	Moderate
Jing et al.[24]	++	+	+	+	+	+	+	8	Low
Kim et al.[25]	++	+	0	0	+	+	0	5	Moderate
Korea CDC[26]	++	0	0	0	0	+	0	3	High
Laxminarayan et al.[27]	+	+	+	+	+	+	0	6	Moderate
Lee et al.[28]	0	+	0	0	+	0	0	2	High
Lewis et al.[29]	+	+	0	+	+	+	+	6	Moderate

© 2020 Madewell ZJ et al. *JAMA Network Open.*

Li et al.[30]	+	+	+	+	+	+	+	7	Low
Liu et al.[31]	++	+	+	+	+	+	+	8	Low
Lopez Bernal et al.[32]	++	+	+	+	0	+	0	6	Moderate
Luo et al.[33]	++	0	+	0	+	+	+	6	Moderate
Lyngse et al.[34]	++	+	+	+	+	+	0	7	Low
Malheiro et al.[35]	++	+	+	0	0	+	0	5	Moderate
Park, Choe et al.[36]	++	+	+	+	+	+	+	8	Low
Park, Kim et al.[37]	0	0	0	+	+	+	0	3	High
Patel et al.[38]	0	+	0	0	0	0	0	1	High
Phiriyasart et al.[39]	0	+	0	0	+	+	+	4	Moderate
Rosenberg et al.[40]	++	+	+	+	+	0	0	7	Low
Shah et al.[41]	+	0	+	0	0	++	0	4	Moderate
Son et al.[42]	++	+	0	0	+	0	0	4	Moderate
Sun et al.[43]	+	0	+	+	0	0	0	3	High
Teherani et al.[44]	+	+	0	+	0	0	0	3	High
van der Hoek et al.[45]	+	0	0	+	+	++	+	6	Moderate
Wang, Ma et al.[46]	+	0	0	+	0	+	0	3	High
Wang, Pan et al.[47]	++	0	+	0	0	+	+	5	Moderate
Wang, Tian et al.[48]	+	+	+	+	+	+	0	6	Moderate
Wang, Zhou et al.[49]	0	+	0	0	0	0	0	1	High
Wu, Huang et al.[50]	+	+	0	+	+	++	+	7	Low
Wu, Song et al.[51]	+	0	0	0	+	+	0	3	High
Xin et al.[52]	++	+	0	+	+	+	+	7	Low
Yu et al.[53]	++	0	+	0	0	+	0	4	Moderate
Yung et al.[54]	+	0	0	+	+	+	0	4	Moderate
Zhang, Cheng et al.[55]	0	+	0	0	+	0	+	3	High
Zhang, Zhou et al.[56]	0	+	0	0	+	0	0	2	High
Zhuang et al.[57]	++	0	+	0	+	0	0	4	Moderate

[a] ++: Representative of COVID-19 cases in region; +: Somewhat representative; 0: Poorly described or not representative of cases in region
[b] +: Index case identified by date of onset of symptoms and/or test dates; 0: First case not clearly defined
[c] +: ≥300 contacts; 0: <300 contacts
[d] +: Secondary attack rate disaggregated by ≥1 covariate; 0: Secondary attack rate not disaggregated by any covariates
[e] +: Tested all contacts (both symptomatic and asymptomatic); 0: Only tested symptomatic contacts
[f] ++: >14 days; +: 14 days; 0: <14 days or not specified
[g] +: ≥2 tests; 0: 1 test or not described
[h] High: ≤3 points; moderate: 4–6 points; low: ≥7 points

eTable 3. Description of Index Cases for Studies Included in Review of Household Transmissibility of SARS-CoV-2

	Location (city, country)	Index case identification period	Index case identification method (passive, active[a] surveillance)	No. index cases	Index case symptom status	Transmission mitigation strategies after index case diagnosis
Adamik et al.[4]	Poland	March to July 1, 2020	Passive	13,309	Symptomatic	Index cases isolated
Arnedo-Pena et al.[5]	Castellon, Spain	February 26 to April 8, 2020	Passive	347	Symptomatic	–
Bae et al.[6]	Cheonan, Korea	February 24 to March 13, 2020	Active	57	Symptomatic	Contacts quarantined
Bi et al.[7]	Shenzhen, China	January 14 to February 12, 2020	Active and passive	391	Symptomatic or asymptomatic	Index cases isolated
Böhmer et al.[8]	Bavaria, Germany	January 27 to February 11, 2020	Passive	–	–	Contacts quarantined
Boscolo-Rizzo et al.[9]	Treviso province, Italy	March 19 to March 22, 2020	Passive	179	Symptomatic	Contacts quarantined
Burke et al.[10]	USA	January 20 to February 26, 2020	Passive	10	Symptomatic	Contacts quarantined
Chaw et al.[11]	Brunei	March 5 to April 4, 2020	Active and passive	19	Symptomatic or asymptomatic	Index cases isolated and contacts quarantined
Chen et al.[12]	Ningbo, China	January 21 to March 6, 2020	Active	157	Symptomatic or asymptomatic	Index cases isolated
Cheng et al.[13]	Taiwan	January 15 to March 18, 2020	Active and passive	100	Symptomatic or asymptomatic	Contacts quarantined
Dattner et al.[14]	Bnei Brak, Israel	May 2, 2020	Passive	637	Symptomatic or asymptomatic	–
Dawson et al.[15]	Milwaukee County, Wisconsin, USA	March to April, 2020	Passive	26	Symptomatic	–
Dong et al.[16]	Tianjin, China	January 7 to February 24, 2020	Active and passive	135	Symptomatic or asymptomatic	–

© 2020 Madewell ZJ et al. *JAMA Network Open.*

Doung-ngern et al.[17]	Thailand	March 1 to 31, 2020	Active and passive	18	Symptomatic	Contacts quarantined
Draper et al.[18]	Northern Territory, Australia	March 1 and April 30, 2020	Passive	28	Symptomatic	Cases isolated
Fateh-Moghadam et al.[19]	Trento, Italy	March to April, 2020	Passive	1,489	Symptomatic	Contacts quarantined
Han et al.[20]	South Korea	March 28, 2020	Active	10	Symptomatic or asymptomatic	Cases isolated
Hu et al.[21]	Hunan, China	January 16 to April 02, 2020	Active and passive	1,178	Symptomatic or asymptomatic	Cases isolated
Hua et al.[22]	Zhejiang Province, China	January 7 to February 29, 2020	Passive	314	Symptomatic or asymptomatic	Cases isolated
Islam et al.[23]	Chattogram, Bangladesh	April 3 to June 2, 2020	Active and passive	181	Symptomatic or asymptomatic	Contacts quarantined
Jing et al.[24]	Guangzhou, China	January 6 to February 17, 2020	Active and passive	349	Symptomatic or asymptomatic	Evaluated scenarios with/without quarantine/isolation
Kim et al.[25]	South Korea	January 20 to April 6, 2020	Passive	107	Symptomatic or asymptomatic	Index cases isolated and contacts quarantined
Korea CDC[26]	South Korea	January 24 to March 10, 2020	Active	30	Symptomatic or asymptomatic	Cases isolated
Laxminarayan et al.[27]	Tamil Nadu and Andhra Pradesh, India	March 5 to June 4, 2020	Active and passive	998	Symptomatic or asymptomatic	–
Lee et al.[28]	Busan, South Korea	February 21 to March 13, 2020	Active and passive	10	Asymptomatic or pre-symptomatic	Cases isolated
Lewis et al.[29]	Wisconsin and Utah, USA	March 22 to April 25, 2020	Passive	58	Symptomatic	–
Li et al.[30]	Wuhan, China	January 1 to February 20, 2020	Passive	105	Symptomatic	Contacts quarantined
Liu et al.[31]	Guangdong Province, China	January 10 to March 15, 2020	Active and passive	1,158	Symptomatic	Cases isolated

© 2020 Madewell ZJ et al. *JAMA Network Open.*

Lopez Bernal et al.[32]	U.K.	January 24 to March 13, 2020	Passive	365	Symptomatic	–
Luo et al.[33]	Guangzhou, China	January 13 to March 6, 2020	Active and passive	69	Symptomatic or asymptomatic	Contacts quarantined
Lyngse et al.[34]	Denmark	February 27 to July 24, 2020	Passive	990	Symptomatic or asymptomatic	–
Malheiro et al.[35]	Eastern Porto, Portugal	March 1, 2020 to April 30, 2020	Active and passive	453	Symptomatic	Cases isolated
Park, Choe et al.[36]	South Korea	January 20 to March 27, 2020	Active and passive	5,706	Symptomatic or asymptomatic	Contacts quarantined
Park, Kim et al.[37]	Seoul, South Korea	February 21 to March 8, 2020		97	Symptomatic or asymptomatic	Index cases isolated and contacts quarantined
Patel et al.[38]	London, UK	March 1 to April 1, 2020	Active and passive	141	Symptomatic	Cases isolateds
Phiriyasart et al.[39]	Pattani Province, Thailand	March 30 to April 20, 2020	Passive	25	Symptomatic	–
Rosenberg et al.[40]	New York, USA	March 2 to March 12, 2020	Active and passive	229	Symptomatic or asymptomatic	–
Shah et al.[41]	Gujarat, India	March 28 to July 2, 2020	Passive	74	Symptomatic	–
Son et al.[42]	Busan, South Korea	January 16 to March 24, 2020	Active and passive	108	Symptomatic or asymptomatic	Contacts quarantined
Sun et al.[43]	Zhejiang Province, China	January 20 to February 10, 2020	Active and passive	148	Symptomatic or asymptomatic	–
Teherani et al.[44]	Atlanta, USA	March 16 to June 14, 2020	Passive	32	Symptomatic	–
van der Hoek et al.[45]	Netherlands	March to April 2, 2020	Passive	54	Symptomatic	–
Wang, Ma et al.[46]	Wuhan, China	February 13 and February 14, 2020	Passive	85	Symptomatic	Contacts quarantined
Wang, Pan et al.[47]	Beijing, China	January 1 to April 3, 2020	Active and passive	585	Symptomatic or asymptomatic	Contacts quarantined
Wang, Tian et al.[48]	Beijing, China	February 21, 2020	Passive	41	Symptomatic	Cases isolated
Wang, Zhou et al.[49]	Wuhan, China	January 5 to 12 February, 2020	Active	25	Symptomatic	Contacts quarantined

© 2020 Madewell ZJ et al. *JAMA Network Open.*

Wu, Huang et al.[50]	Zhuhai, China	January 17 to February 29, 2020	Active and passive	35	Symptomatic or asymptomatic	Cases isolated
Wu, Song et al.[51]	Hangzhou, China	January 23 to February 28, 2020	Active and passive	144	Symptomatic or asymptomatic	Cases isolated
Xin et al.[52]	Qingdao Municipal, China	January 20 to March 27, 2020	Active and passive	31	Symptomatic or asymptomatic	Cases isolated
Yu et al.[53]	Wuhan, China	January 14 to February 14, 2020	–	560	Symptomatic	Contacts quarantined
Yung et al.[54]	Singapore	March 5 to April 30, 2020	Passive	223	Symptomatic	Cases isolated and contacts quarantined
Zhang, Cheng et al.[55]	China	January 28 to March 15, 2020	Active	359	Symptomatic or asymptomatic	Contacts quarantined
Zhang, Zhou et al.[56]	Liaocheng, China	January 13 to 26, 2020	Active	11	Symptomatic or asymptomatic	Cases isolated
Zhuang et al.[57]	Guangdong Province, China	January 1 to February 29, 2020	Passive	283	Symptomatic	–

[a]Active surveillance: (e.g., travelers from areas with active SARS-CoV-2 transmission; individuals detected by neighborhood fever screenings).

© 2020 Madewell ZJ et al. *JAMA Network Open.*

eTable 4. Description of Contacts for Studies Included in Review of Household Transmissibility of SARS-CoV-2

	Contact type (household, family)	Test used to diagnosis contacts	Universal testing or only symptomatic	Number of tests per contact	Follow up duration (days)	Household/family overall secondary attack rate	Close contact secondary attack rate
Adamik et al.[4]	Household	–	Symptomatic	–	–	11.1% (3,553/32,023)	–
Arnedo-Pena et al.[5]	Household	Symptom-based diagnosis (no testing of contacts)	Symptomatic	0	–	11.1% (83/745)	–
Bae et al.[6]	Household	RT-PCR	Symptomatic	1	14	21.6% (37/200)	6.4% (108/1,687)
Bi et al.[7]	Household	RT-PCR	Symptomatic and asymptomatic	1	14	11.2% (77/686)	7.6% (98/1,286)
Böhmer et al.[8]	Household	RT-PCR and whole genome sequencing	Symptomatic and asymptomatic	Multiple	14	20.8% (5/24)	6.6% (16/241)
Boscolo-Rizzo et al.[9]	Household	RT-PCR	Symptomatic	1	14	43.0% (54/121)	–
Burke et al.[10]	Household	–	Symptomatic	1	14	10.5% (2/19)	0.4% (2/445)
Chaw et al.[11]	Household	RT-PCR	Symptomatic and asymptomatic	Tested all contacts once; extra tests for symptomatic	14	10.6% (28/264)	2.9% (51/1,755)
Chen et al.[12]	Family	Nucleic acid test	Symptomatic and asymptomatic	Multiple	21	18.0% (49/272)	6.1% (132/2,147)
Cheng et al.[13]	Household	RT-PCR	Symptomatic	Tested all contacts once; extra tests for symptomatic	14	6.6% (10/151)	0.8% (22/2,761)
Dattner et al.[14]	Household	RT-PCR	Symptomatic	Tested all contacts once; extra tests for symptomatic	–	34.7% (981/2824)	–

© 2020 Madewell ZJ et al. *JAMA Network Open.*

Dawson et al.[15]	Household	RT-PCR	Symptomatic and asymptomatic	Tested all contacts once; extra tests for symptomatic	–	25.0% (16/64)	–
Dong et al.[16]	Family	RT-PCR	Symptomatic and asymptomatic	Multiple	–	20.5% (53/259)	–
Doung-ngern et al.[17]	Household	RT-PCR	Asymptomatic	1	21	16.5% (38/230)	–
Draper et al.[18]	Household	RT-PCR	Symptomatic	1	14	3.9% (2/51)	0.9% (4/445)
Fateh-Moghadam et al.[19]	Household	RT-PCR	Symptomatic	1	14	14.1% (500/3,546)	13.4% (840/6,255)
Han et al.[20]	Household	RT-PCR	Symptomatic and asymptomatic	Tested all contacts once; extra tests for symptomatic	14	21.4% (3/14)	19.4% (7/36)
Hu et al.[21]	Household	RT-PCR	Symptomatic and asymptomatic	1	14	17.7% (491/2771)	3.0% (471/15,648)
Hua et al.[22]	Family	RT-PCR	Symptomatic and asymptomatic	1	–	18.1% (151/835)	–
Islam et al.[23]	Household	–	Symptomatic and asymptomatic	1	14	13.0% (6/46)	–
Jing et al.[24]	Household	RT-PCR or sequencing	Symptomatic and asymptomatic	Multiple	14	17.1% (93/542)	–
Kim et al.[25]	Household	RT-PCR	Symptomatic and asymptomatic	1	14	0.5% (1/208)	–
Korea CDC[26]	Household	–	Symptomatic	1	14	7.6% (9/119)	0.5% (13/2,370)
Laxminarayan et al.[27]	Household	RT-PCR	Symptomatic and asymptomatic	1	14	9.0% (380/4065)	13.4% (623/4,637)
Lee et al.[28]	Household	RT-PCR	Symptomatic and asymptomatic	1	–	4.3% (1/23)	–

© 2020 Madewell ZJ et al. *JAMA Network Open.*

Lewis et al.[29]	Household	RT-PCR	Symptomatic and asymptomatic	Tested all contacts once; extra tests for symptomatic	14	29.3% (55/188)	–
Li et al.[30]	Household	RT-PCR	Symptomatic and asymptomatic	Tested all contacts once; extra tests for symptomatic	14	16.3% (64/392)	–
Liu et al.[31]	Family	RT-PCR	Symptomatic and asymptomatic	Multiple	14	13.5% (330/2441)	4.4% (515/11,580)
Lopez Bernal et al.[32]	Household	RT-PCR	Symptomatic	1	14	34.1% (161/472)	–
Luo et al.[33]	Household	RT-PCR	Symptomatic and asymptomatic	Multiple	14	10.2% (105/1015)	3.7% (127/3,410)
Lyngse et al.[34]	Household	–	Symptomatic and asymptomatic	1	14	16.7% (371/2226)	–
Malheiro et al.[35]	Household	RT-PCR	Symptomatic	1	14	10.5% (72/685)	9.5% (154/1,627)
Park, Choe et al.[36]	Household	RT-PCR	Symptomatic and asymptomatic	Multiple	14	11.8% (1,248/10,592)	3.7% (2169/59,073)
Park, Kim et al.[37]	Household	RT-PCR	Symptomatic and asymptomatic	1	14	15.1% (34/225)	–
Patel et al.[38]	Household	COVID-19 symptoms (no testing)	Symptomatic	0	–	43.0% (79/185)	–
Phiriyasart et al.[39]	Household	RT-PCR	Symptomatic and asymptomatic	Additional tests after initial negative	14	11.3% (12/106)	6.5% (25/387)
Rosenberg et al.[40]	Household	Molecular SARS-CoV-2 tests	Symptomatic and asymptomatic	1	–	38.0% (131/343)	–
Shah et al.[41]	Household	RT-PCR	Symptomatic	1	28	8.8% (34/386)	–

© 2020 Madewell ZJ et al. *JAMA Network Open.*

Son et al.[42]	Household	RT-PCR	Symptomatic and asymptomatic	1	–	8.2% (16/196)	–
Sun et al.[43]	Household	–	Symptomatic	1	–	31.6% (189/598)	–
Teherani et al.[44]	Household	Symptom-based diagnosis (no testing)	Symptomatic	0	–	28.7% (31/108)	–
van der Hoek et al.[45]	Family	RT-PCR and serological diagnostics	Symptomatic and asymptomatic	Multiple	21	27.0% (47/174)	–
Wang, Ma et al.[46]	Household	RT-PCR	Symptomatic	1	14	30% (47/155)	–
Wang, Pan et al.[47]	Household	RT-PCR, gene sequencing	–	Tested all contacts once; extra tests for symptomatic	14	15.6% (111/714)	4.6% (186/4,007)
Wang, Tian et al.[48]	Household	RT-PCR, gene sequencing	Symptomatic and asymptomatic	1	14	23.0% (77/335)	–
Wang, Zhou et al.[49]	Family	Nucleotide tests	Symptomatic		–	23.3% (10/43)	–
Wu, Huang et al.[50]	Household	RT-PCR	Symptomatic and asymptomatic	Multiple	21	32.4% (48/148)	–
Wu, Song et al.[51]	Household	–	Symptomatic and asymptomatic	1	14	17.9% (50/280)	2.7 (82/2,994)
Xin et al.[52]	Household	RT-PCR	Symptomatic and asymptomatic	Multiple	14	17.9% (19/106)	–
Yu et al.[53]	Family	–	–	1	14	10.2% (143/1396)	9.5% (150/1,587)
Yung et al.[54]	Household	RT-PCR	Symptomatic and asymptomatic	1	14	6.1% (13/200)	–
Zhang, Cheng et al.[55]	Household	Nucleic acid tests	Symptomatic and asymptomatic	Tested all contacts once; extra	–	16.1% (10/62)	3.3% (12/369)

© 2020 Madewell ZJ et al. *JAMA Network Open.*

				tests for symptomatic			
Zhang, Zhou et al.[56]	Family	RT-PCR	Symptomatic and asymptomatic	1	–	12.9% (12/93)	–
Zhuang et al.[57]	Family	–	Symptomatic and asymptomatic	1	–	7.5% (276/3697)	2.9% (239/8,319)

© 2020 Madewell ZJ et al. *JAMA Network Open.*

eTable 5. Overdispersion of the Number of Secondary Infections of SARS-CoV-2 per Household

Author	Location	Average number of contacts per household	Secondary attack rate	Proportion of households reporting any secondary transmission from index cases	Probability of ≥1 secondary infection in a household[a]
Wu, Huang et al.	Zhuhai, China	4.229	0.324	0.629	0.809
Rosenberg et al.	New York, USA	3.330	0.382	0.612	0.799
Lewis et al.	Utah & Wisconsin, USA	3.241	0.277	0.552	0.650
Wang, Tian et al.	Beijing, China	2.702	0.230	0.331	0.506
Shah et al.	Gujarat, India	5.216	0.088	0.216	0.382
Yung et al.	Singapore	1.493	0.065	0.052	0.095
Draper et al.	Northern Territory, Australia	1.821	0.039	0.036	0.070

[a]To examine for potential over-dispersion, we crudely assume that all households have size equal to the average number of contacts and equal secondary attack rate. Then, $\mathrm{Pr}[\geq 1\ \text{secondary infection in a household}] = 1-(1-\mathrm{SAR})^{\mathrm{n}}$, where n is the average number of contacts for that study. This is a crude analysis that does not consider heterogeneity in household size. Given this limitation, statistical significance is not assessed, with results intended for illustration only.

© 2020 Madewell ZJ et al. *JAMA Network Open.*

eTable 6. Assessment of Factors Potentially Affecting Susceptibility and Infectivity of SARS-CoV-2 in Household Transmission Studies

Risk factors	Overall number of studies	Number of studies reporting significant associations
**Susceptibility (of contacts)**		
Age	23	14
Sex	19	3
Exposure period (to index case)	7	3
Relationship	6	4
Underlying medical conditions	1	1
BMI	1	0
Comorbidity	1	0
Educational attainment	1	0
Race/ethnicity	1	0
**Infectivity (of index case)**		
Clinical severity	9	6
Age	9	3
Sex	9	3
Cough	8	2
Fever	6	0
Diarrhea	3	1
Myalgia	3	1
Comorbidity	3	0
Fatigue	3	0
Hospitalization	2	2
Pneumonia	2	2
Expectoration	2	1
Lymphocyte count	2	1
Educational attainment	2	0
Acute respiratory distress syndrome/sepsis	1	1
Chills	1	1
Comorbidity	1	1
Dizziness	1	1
Health profession	1	1
Neutrophil percentage	1	1

© 2020 Madewell ZJ et al. *JAMA Network Open.*

Abdominal pain	1	0
Arthralgia	1	0
BMI	1	0
Chest tightness	1	0
Dyspnea	1	0
Headache	1	0
Nasal congestion	1	0
Nausea	1	0
Palpitation	1	0
Pharyngalgia	1	0
Poor appetite	1	0
Race/ethnicity	1	0
Rhinorrhea	1	0
Vomiting	1	0
White blood cell count	1	0
**Awareness and behavioral factors**		
Contact frequency	6	5
Shared vehicle (with index case)	6	5
Contacts wore face masks	4	4
Shared meal	3	2
Self-isolated after illness onset	3	1
Wear mask at home after illness onset	3	1
Time interval from illness onset to hospital admission	3	0
Physical contact	2	2
Disinfectant use[a]	2	1
Ventilation duration[a]	2	1
Contacts smoking status	2	0
Index case eats with separate tableware	2	0
Index case smoking status	2	0
Time interval from illness onset to laboratory confirmation	2	0
Contacts hand hygiene	1	1
Knowledge of own infectiousness after illness onset	1	1
Shared cigarette	1	1
Shared living room	1	1
Frequency of room cleaning (wet type)	1	0
Index case hand hygiene	1	0
Index case slept in separate bedroom	1	0
Index case used private bathroom	1	0

© 2020 Madewell ZJ et al. *JAMA Network Open.*

Knowledge score on COVID-19 before illness onset	1	0
Self-awareness of being infected with SARS-CoV-2 when developed illness	1	0
Time interval from illness onset to medical isolation	1	0
**Household characteristics**		
Number of people in household	7	4
Number of washrooms	2	0
Residential area per person	2	0
Number of bedrooms per person	1	0
Separate dining area	1	0
Urban/rural residence	1	0
[a] Includes one study that combined ventilation and disinfection, which was not significant		

© 2020 Madewell ZJ et al. *JAMA Network Open.*

eTable 7. Household Secondary Attack Rate Comparison With Other Viruses

	Location	Number of index cases	Overall household secondary attack rate	Definition of household contact	Case ascertainment
**Middle East Respiratory Syndrome**					
Al Hosani et al.[58]	Abu Dhabi, United Arab Emirates	34	0% (0/105)	Household contacts	RT-PCR
Arwady et al.[59]	Al-Qouz, Saudi Arabia	5	24% (19/79)	Family contacts	RT-PCR
Assiri et al.[60]	Al-Hasa, Saudi Arabia	23	2.3% (5/217)	Household contacts	RT-PCR
Drosten et al.[61]	Saudi Arabia	26	4% (12/280)	Household contacts	RT-PCR
Memish et al.[62]	Al-Madinah al-Munawwarah, Saudi Arabia	18	1.8% (1/56)	Household contacts	RT-PCR
Payne et al.[63]	Jordan	16	9% (2/23)	Household contacts	RT-PCR
Van Kerkhove et al.[64]	Riyadh, Saudi Arabia	7	5.1% (12/234.5)	Household contacts	RT-PCR
**SARS-CoV**					
Chan et al.[65]	Hong Kong, China	99	12.3% (30/243)	Household contacts	Positive contacts identified by the Hong Kong SARS registry
Goh et al.[66]	Singapore	114	6.2% (26/417)	Household contacts	PCR or serologic test
Lau et al.[67]	Hong Kong, China	881	8% (188/2139)	Household contacts	Symptomatic probable cases (no testing)
Ou et al.[68]	Beijing, China	232	3.3% (18/550)	Household contacts	–
Pang et al.[69]	Beijing, China	582	11.4% (111/973)	Household contacts	Symptom-based diagnosis (no testing)
Tuan et al.[70]	Vietnam	45	2.4% (3/123)	Household contacts	RT-PCR
Wilson-Clark et al.[71]	Toronto, Canada	74	10.2% (18/176)	Household contacts	Symptom-based diagnosis (no testing)
**Other coronaviruses**					
Beale et al.[72]	England, UK	70 HCoV-OC43<br>45 HCoV-229E<br>55 HCoV-NL63	HCoV-OC43: 13.2% (7/53)<br>HCoV-229E: 10.1% (7/69)<br>HCoV-NL63:11.9% (5/42)	Household contacts	RT-PCR
Esposito et al.[73]	Milan, Italy	33 HCoV-229E<br>13 HCoV-NL63<br>11 HCoV-OC43	HCoV-229E: 14.9% (14/94)<br>HCoV-NL63: 0% (0/45)<br>HCoV-OC43: 13.2% (5/38)	Household contacts	RT-PCR

© 2020 Madewell ZJ et al. *JAMA Network Open.*

Monto et al.[74]	Michigan, USA	263 HCoV-OC43<br>113 HCoV-229E<br>141 HCoV-HKU1<br>217 HCoV-NL63	HCoV-OC43: 10.6%<br>HCoV-229E: 7.2%<br>HCoV-HKU1: 8.6%<br>HCoV-NL63:12.6%	Household contacts	RT-PCR
**Influenza**					
Tseng et al.[1]	Review of household transmission studies		SAR ranged from 1–38% based on PCR-confirmed infection, 6–35% based on influenza-like illness, and 3–31% based on acute respiratory illness		

© 2020 Madewell ZJ et al. *JAMA Network Open.*

eTable 8. Weights for Combined Estimate of Secondary Attack Rates of Severe Acute Respiratory Syndrome Coronavirus 2 (SARS-CoV-2) for Household Contacts and Family Contacts

Authors, Location	Weight
Boscolo−Rizzo et al,[9] Treviso Province, Italy	1.53%
Patel et al,[38] London, UK	1.73%
Rosenberg et al,[40] New York, USA	1.97%
Dattner et al,[14] Bnei Brak, Israel	2.27%
Lopez Bernal et al,[32] U.K.	2.06%
Wu, Huang et al,[50] Zhuhai, China	1.66%
Sun et al,[43] Zhejiang Province, China	2.11%
Wang, Ma et al,[46] Wuhan, China	1.69%
Teherani et al,[44] Atlanta, USA	1.52%
Lewis et al,[29] Utah & Wisconsin, USA	1.78%
van der Hoek et al,[45] Netherlands	1.75%
Dawson et al,[15] Wisconsin, USA	1.25%
Wang, Zhou et al,[49] Wuhan, China	1.04%
Wang, Tian et al,[48] Beijing, China	2.00%
Han et al,[20] South Korea	0.50%
Böhmer et al,[8] Bavaria, Germany	0.74%
Dong et al,[16] Tianjin, China	1.94%
Bae et al,[6] Cheonan, Korea	1.86%
Hua et al,[22] Zhejiang Province, China	2.19%
Chen et al,[12] Ningbo, China	1.97%
Xin et al,[52] Qingdao Municipal, China	1.59%
Wu, Song et al,[51] Hangzhou, China	1.98%
Hu et al,[21] Hunan, China	2.27%
Jing et al,[24] Guangzhou, China	2.13%
Lyngse et al,[34] Denmark	2.27%
Doung−ngern et al,[17] Thailand	1.92%
Li et al,[30] Wuhan, China	2.07%
Zhang, Cheng et al,[55] China	1.33%
Wang, Pan et al,[47] Beijing, China	2.18%
Park, Kim et al,[37] Seoul, South Korea	1.93%
Fateh−Moghadam et al,[19] Trento, Italy	2.29%
Liu et al,[31] Guangdong Province, China	2.27%
Islam et al,[23] Chattogram, Bangladesh	1.21%
Zhang, Zhou et al,[56] Liaocheng, China	1.59%
Park, Choe et al,[36] South Korea	2.31%
Phiriyasart et al,[39] Pattani Province, Thailand	1.68%
Bi et al,[7] Shenzhen, China	2.19%
Arnedo−Pena et al,[5] Castellon, Spain	2.20%

© 2020 Madewell ZJ et al. *JAMA Network Open.*

Adamik et al,[4] Poland	2.31%
Malheiro et al,[35] Eastern Porto, Portugal	2.20%
Chaw et al,[11] Brunei	2.02%
Burke et al,[10] USA	0.77%
Luo et al,[33] Guangzhou, China	2.23%
Yu et al,[53] Wuhan, China	2.25%
Laxminarayan et al,[27] TN & AP, India	2.29%
Shah et al,[41] Gujarat, India	2.12%
Son et al,[42] Busan, South Korea	1.97%
Korea CDC,[26] South Korea	1.81%
Zhuang et al,[57] Guangdong Province, China	2.29%
Cheng et al,[13] Taiwan	1.92%
Yung et al,[54] Singapore	2.00%
Lee et al,[28] Busan, South Korea	1.10%
Draper et al,[18] Northern Territory, Australia	1.57%
Kim et al,[25] South Korea	2.22%
	100%

© 2020 Madewell ZJ et al. *JAMA Network Open.*

eAppendix 1. Eligibility Criteria

We included articles with original data for estimating household secondary attack rate of SARS-CoV-2. The publication must report at least two of numerator, denominator, and secondary attack rate among household contacts. Where numerators (numbers of infected/sick contacts) or denominators (numbers of contacts) were not reported but the number of index cases and secondary attack rate were available, the denominator was calculated acknowledging limits of significant digits. We did not contact authors for additional data.

Household transmission of SARS-CoV-2 is described in case reports in narrative form of individual families or households with large numbers of cases, and contact tracing investigations whereby investigators identify the number of infected household or family members from index cases, usually over a period of 14 or 21 days. We excluded studies that 1) were case reports of individual families or households as these can bias results towards high attack rates, 2) reported infection prevalence in the household without describing transmission, 3) tested household contacts using antibody tests as antibodies may be detected many weeks after infection,[75] 4) were of close contacts that did not report secondary attack rates for household or family members, 5) were preprints of published articles already included in the review, and 6) had overlapping populations with another study already included in the review. We used the same eligibility criteria for studies of other coronaviruses. One reviewer first screened studies by titles and abstracts to identify potential studies for inclusion. One reviewer subsequently evaluated full-text articles and selected those that met the inclusion criteria.

© 2020 Madewell ZJ et al. *JAMA Network Open.*

eAppendix 2. Data Extraction

One reviewer extracted the following information for studies of SARS-CoV-2: first author, location, index case identification period, index case identification method, number of index cases, index case symptom status, transmission mitigation strategies, household/family/close contact type, test used to diagnose contacts, universal/symptomatic testing, number of tests per contact, follow-up duration, number of infected household contacts, total number of household contacts, household secondary attack rate, and transmission risk factors. For studies that reported secondary attack rates for both household and family contacts, only the household contact secondary attack rate was extracted. For studies that included secondary attack rates for close contacts, we also extracted the number of infected close contacts, total number of close contacts, and close contact secondary attack rate.

For studies of other coronaviruses, one reviewer extracted: first author, location, number of index cases, number of infected contacts, total number of contacts, secondary attack rate, contact type, and test used to diagnose contacts. For influenza, we extracted secondary attack rate ranges reported in the review.

The following methods describe how secondary attack rates were calculated and how specific covariates were categorized if there was ambiguity during the process of data extraction:

SARS-CoV-2

- *Bi et al.*[7]: Contact age was reported in increments of ten years (0-9, 10-19,…), which we categorized into 0-19 and ≥20 years (child category therefore includes 18 and 19 years).
- *Böhmer et al.*[8]: SAR reported as 75.0% (95% CI 19.0–99.0%; 3/4) among members of a household cluster in common isolation and 10.0% (1.2–32.0%; 2/20) among household contacts only together until isolation of the patient, so overall SAR=5/24=20.8%.
- *Chaw et al.*[11]: There were 12 infections among 85 children, 13 infections among 31 spouses, and 3 infections among 148 other household contacts, so overall household SAR=28/264=10.6%, children SAR=12/85=14.1%, and adult SAR=16/179=8.9%.
- *Chen et al.*[12]: Family SAR (18.01%) and total number of family members (272) were provided, so the number of infected family members=.1801*272=49.
- *Cheng et al.*[13]: Total number of household secondary cases (10) and number of household contacts (151) were provided, so overall household SAR=10/151=6.6%. Contact age was reported as 0-19; 20-39; 40-59; ≥60, which we categorized into 0-19 years and ≥20 years (children category includes 18 and 19 years).
- *Dattner et al.*[14]*:* This study reported a total of 1544 children including index cases, 512 of which were infected (33%). Excluding the index cases, they reported that 25% of children were infected, but did not provide the numerator or denominator. Therefore, we estimated the number of index cases, secondary infections, and total contacts by solving for x: (512-x)/(1544-x)=.25; x=168 index cases; 512-168=344 secondary infections among children; 1544-168=1376 total contacts. This study also reported a total of 1809 adults including index cases, 998 of which were infected (55%). Excluding the index cases, they reported 44% of adults were infected, but did not provide the numerator or denominator. Solving again for x, we estimate there were: (998-x)/(1809-x)=.44; x=361 index cases; 998-361=637 secondary infections among adults; 1809-361=1448 total contacts. The total number of infected secondary infections=344+637=981, the total number of contacts=1376+1448=2824, and overall SAR=981/2824=34.7%.
- *Draper et al.*[18]: This study reported 2 infections among 51 total household contacts, both from the same household. The total number of households were not reported, but since there were 28 total index cases, we assumed there were 28 households and 1/28 households (3.6%) had secondary infections.
- *Hu et al.*[21]: Number of household contacts (2,771) and SAR (17.7%) provided, so number of infected contacts=491.
- *Hua et al.*[22]: This study reported 151 total infections and 684 negative results, so overall SAR=151/835=18.1%.
- *Jing et al.*[24]: We calculated the total number of household contacts at the same residential address as the number of household secondary cases (93) plus the number of uninfected household contacts (449), which is 542. The total number of female contacts = 53 infected + 227 uninfected = 280; total male contacts = 40 infected + 218 uninfected = 258. Total contacts <20 years = 8 infected + 117 uninfected = 125 total children (includes 18 and 19 years). We collapsed adults into ≥20 years: there were 85 infected + 327 uninfected = 412 total.

- *Kim et al.*[25]: Of 248 household contacts, 41 had COVID-19, but 40 had the same exposure as the index case, so SAR=1/208=0.5%.
- *Liu et al.*[31]: There were 131 infections among 563 total spouses and 199 infections among 1878 non-spouse family members, so overall SAR = (131+199)/(563+1878) = 330/2441 = 13.5%. Contact age was reported as 0-9 years, 10-19 years, …, which we collapsed to 0-19 and ≥20 categories (children category includes 18 and 19 years).
- *Lopez Bernal et al.*[32]: The article reported the household SAR as 37% for probable and confirmed cases without providing a numerator or denominator. Table 1 indicated there were 96 probable cases and 65 confirmed cases=161 total cases out of 472 household contacts, which=34.1%. Age was reported in Table 1 as <19, 19-64, ≥65, which we categorized into 0-18 and ≥19 years (children category includes 18 years).
- *Malheiro et al.*[35]*:* The SARs for the intervention and control groups were 11.6% (11/95) and 10.5% (72/685). Therefore, the overall SAR=83/780=10.6%.
- *Park, Kim et al.*[37]: This article reported 17 contacts of asymptomatic index patients, but Table 1 reported 15 total (4 asymptomatic and 11 pre-symptomatic). We used 15 contacts in our analysis as depicted in the table as the more conservative estimate.
- *Teherani et al.*[44]: Of 144 (58 children; 86 adults) household contacts, 67 developed symptoms including 31 (11 children; 20 adults) after onset of symptoms in the index case and 36 (14 children; 22 adults) before symptom onset in the index case. Therefore, the SAR from index cases=31/108=28.7% (child contact SAR=11/44=25.0%; adult contact SAR=20/64=31.3%).
- *Wu, Huang et al.*[50]: Contact age was reported as 0-3, 4-18, 19-60, and >61 years categories, which we collapsed into ≤18 and >18 categories (children category includes 18 years).
- *Yung et al.*[54]: Contact age reported as 0-4; 5-9; 10-16 years (children category does not include 17 years).

Other viruses:

- *Monto et al.*[74]: This study reported overall SARs for HCoV-NL63, HCoV-OC43, HCoV-229E, and HCoV-HKU1 for the entire study period, but did not report the overall numerator and denominator for the entire study period.
- *Ou et al.*[68]: This study reported SARs for household members who contacted the index case during the symptomatic period (SAR: 4.6%; 383 total) and during the incubation period (SAR: 0%; 167 total), so overall SAR=18/550=3.3%.
- *Pang et al.*[69]: There were 338 spouse contacts with a secondary attack rate of 15.4%, so the number of infected spouses=52. There were 635 other household contacts with a secondary attack rate of 8.8% so the number of other infected household members=59. Overall secondary attack rate=111/973=11.4%.
- *Van Kerkhove et al.*[64]: There were 828 total workers in 24 villas. If each villa was considered a house, there were 828/24=34.5 workers per house. There were 19 infected workers in 7 villas. Without the index cases, there were 19-7=12 infected contacts and 33.5*7=235 total household contacts. SAR=12/235=5.1%.

© 2020 Madewell ZJ et al. *JAMA Network Open.*

eAppendix 3. Additional Description of Studies

Household index cases were identified by passive surveillance, active surveillance of key populations (e.g., travelers from areas with active SARS-CoV-2 transmission; individuals detected by neighborhood fever screenings), and both active and passive surveillance (see eTable 3). Some studies included index cases with SARS-CoV-2 infections (both symptomatic and asymptomatic), whereas others included symptomatic COVID-19 index cases only. Another targeted asymptomatic SARS-CoV-2 infected index cases,[28] some of whom developed symptoms during a follow-up period. Several studies stated they assumed all secondary cases were infected by the index case to whom they were traced,[27,36,50] others excluded secondary cases if they developed symptoms before exposure to the primary case,[13,32] another excluded household contacts assessed to have the same exposure as the COVID-19 index cases,[25] and another randomly selected one index case as the infector.[7] Ignoring tertiary transmission inflates the secondary attack rate.

Many studies only included household contacts, but others included family members, or other close contacts, including individuals outside the household. We assumed that studies of household contacts included anyone living in the same household as the index case unless stated otherwise. For example, several studies reported household contacts as family members in households.[6,20,47,48,55] Several studies further restricted household contacts to those who spent at least one night or 24 hours in the house after symptom onset of the index case.[6,25,30,48,50]

Most studies involved tracing contacts and monitoring them for 14 or 21 days. Monitoring methods included phone calls,[6,10,18,22,26,32,35,41] text messages,[10,18] or direct observation by healthcare workers.[33,36] Some studies tested all contacts immediately after the index case was diagnosed at the onset of the observation period and monitored them for symptoms.[7,11,15,20,25,28,54] Others tested all contacts during or at the end of the observation period regardless of symptoms,[17,27,34,37,48,51] whereas others only tested symptomatic contacts (see eTable 4). One study only tested asymptomatic contacts.[17] Several studies tested contacts multiple times throughout the observation period irrespective of symptoms. Of those studies, several also reported extra testing of individuals who developed symptoms during quarantine.[11,15,29,30,47,55] Other studies tested all contacts,[15,21,39,40,43,56] or interviewed index cases about symptoms of household members,[5,38] immediately without additional monitoring. Many studies, particularly those in China, reported in-home quarantine of contacts during the observation period after index cases were confirmed.

Case ascertainment was primarily done via RT-PCR on nasopharyngeal or oropharyngeal samples. Several studies also reported whole-genome sequencing,[8,47-49] and nucleic acid tests.[12,49,55] Three studies identified index cases via RT-PCR, but only collected symptom information about household contacts from telephone interviews with index cases.[5,38,44]

© 2020 Madewell ZJ et al. *JAMA Network Open.*

eAppendix 4. Additional Description of Risk Factors

Factors for infectiousness

Regarding the timing of transmission, several studies reported SARS-CoV-2 transmission prior to index case symptom onset.[13,24,31,55] One study reported higher transmission risk after symptom onset relative to the incubation period.[31] Others found no significant difference in secondary transmission between contacts exposed to index cases before or after illness onset,[13,30,50] although close contacts were quarantined for two studies,[13,30] and index cases were isolated after symptom onset in the third.[50] Some studies reported infection risk peaked during exposure to the index case 2–4 days before or within 5 days of symptom onset.[13,21,31] Another reported a higher secondary attack rate among close contacts of pre-symptomatic index cases than asymptomatic carriers.[13]

Diarrhea,[48] pneumonia,[5,13] acute respiratory distress syndrome,[13] myalgia,[31] chills,[31] dizziness,[31] lymphocyte count,[52] neutrophil percentage,[52] and expectoration,[33] were associated with secondary transmission in some studies. Symptoms not shown to be associated with infectivity were fever,[30,31,33,48,50,52] fatigue,[31,33,52] dyspnea,[31] headache,[31] nasal congestion,[31] pharyngalgia,[31] arthralgia,[31] rhinorrhea,[31] nausea,[31] vomiting,[31] chest tightness,[31] palpitation,[31] poor appetite,[31] abdominal pain,[31] and white blood cell count.[52]

Awareness and behavioral factors

Several studies explored whether prevention measures were associated with reduced transmission. Contacts who wore face masks and index cases who wore masks all the time after illness onset had lower odds of infection and transmission, respectively.[17,48,51] Conversely, contacts who did not apply protective measures (e.g., face mask, avoiding contact with index case) had higher odds of infection.[50] One study reported that greater frequency of chlorine/ethanol based disinfectant use for house cleaning and ventilation hours per day were associated with reduced risk,[48] whereas another did not.[50] Frequency of room cleaning (wet type) and hand hygiene were not significant.[17,48] Index case isolation after illness onset was associated with reduced secondary transmission.[30] Other studies did not find the time interval from illness onset to medical isolation,[48] hospital admission,[48,50,52] or laboratory confirmation,[48,52] to be associated with transmission. Self-awareness of being infected with SARS-CoV-2 and knowledge of COVID-19 were not significant,[48] but lack of knowledge of index case's own infectiousness was associated with transmission.[48] Health profession of the index case was a protective factor in one study.[5]

Physical contact,[17,50] and sharing a vehicle,[7,31,33,50,51] living room,[50] cigarette,[17] or meal,[48,50] were associated with infection, but eating with separate tableware was not.[17,48] Smoking behavior in index cases or contacts was not associated with transmission.[29,50]

© 2020 Madewell ZJ et al. *JAMA Network Open.*

eReferences

1. Tsang TK, Lau LLH, Cauchemez S, Cowling BJ. Household Transmission of Influenza Virus. *Trends in Microbiology.* 2016;24(2):123-133.
2. Wells G. The Newcastle-Ottawa Scale (NOS) for assessing the quality of nonrandomised studies in meta-analysis. *http://www.ohri.ca/programs/clinical_epidemiology oxford htm.* 2004.
3. Fung HF, Martinez L, Alarid-Escudero F, et al. The household secondary attack rate of SARS-CoV-2: A rapid review. *Clinical Infectious Diseases.* 2020.
4. Adamik B, Bawiec M, Bezborodov V, et al. Bounds on the total number of SARS-CoV-2 infections: The link between severeness rate, household attack rate and the number of undetected cases. 2020.
5. Arnedo-Pena A, Sabater-Vidal S, Meseguer-Ferrer N, et al. COVID-19 secondary attack rate and risk factors in household contacts in Castellon (Spain): Preliminary report. *Enfermedades Emergentes.* 2020;19(2):64-70.
6. Bae S, Kim H, Jung T-Y, et al. Epidemiological Characteristics of COVID-19 Outbreak at Fitness Centers in Cheonan, Korea. *J Korean Med Sci.* 2020;35(31).
7. Bi Q, Wu Y, Mei S, et al. Epidemiology and transmission of COVID-19 in 391 cases and 1286 of their close contacts in Shenzhen, China: a retrospective cohort study. *The Lancet Infectious Diseases.* 2020.
8. Böhmer MM, Buchholz U, Corman VM, et al. Investigation of a COVID-19 outbreak in Germany resulting from a single travel-associated primary case: a case series. *The Lancet Infectious Diseases.* 2020.
9. Boscolo-Rizzo P, Borsetto D, Spinato G, et al. New onset of loss of smell or taste in household contacts of home-isolated SARS-CoV-2-positive subjects. *European Archives of Oto-rhino-laryngology.* 2020:1-4.
10. Burke RM. Active monitoring of persons exposed to patients with confirmed COVID-19—United States, January–February 2020. *MMWR Morbidity and mortality weekly report.* 2020;69.
11. Chaw L, Koh WC, Jamaludin SA, Naing L, Alikhan MF, Wong J. SARS-CoV-2 transmission in different settings: Analysis of cases and close contacts from the Tablighi cluster in Brunei Darussalam. *medRxiv.* 2020.
12. Chen Y, Wang AH, Yi B, et al. [Epidemiological characteristics of infection in COVID-19 close contacts in Ningbo city]. *Zhonghua Liu Xing Bing Xue Za Zhi.* 2020;41(5):667-671.
13. Cheng HY, Jian SW, Liu DP, Ng TC, Huang WT, Lin HH. Contact Tracing Assessment of COVID-19 Transmission Dynamics in Taiwan and Risk at Different Exposure Periods Before and After Symptom Onset. *JAMA Internal Medicine.* 2020.
14. Dattner I, Goldberg Y, Katriel G, et al. The role of children in the spread of COVID-19: Using household data from Bnei Brak, Israel, to estimate the relative susceptibility and infectivity of children. *medRxiv.* 2020.
15. Dawson P, Rabold EM, Laws RL, et al. Loss of Taste and Smell as Distinguishing Symptoms of COVID-19. *Clinical Infectious Diseases.* 2020.
16. Dong XC, Li JM, Bai JY, et al. [Epidemiological characteristics of confirmed COVID-19 cases in Tianjin]. *Zhonghua Liu Xing Bing Xue Za Zhi.* 2020;41(5):638-641.
17. Doung-ngern P, Suphanchaimat R, Panjagampatthana A, et al. Case-Control Study of Use of Personal Protective Measures and Risk for Severe Acute Respiratory Syndrome Coronavirus 2 Infection, Thailand. *Emerging Infectious Diseases.* 2020;26(11).
18. Draper AD, Dempsey KE, Boyd RH, et al. The first 2 months of COVID-19 contact tracing in the Northern Territory of Australia, March-April 2020. *Communicable Diseases Intelligence.* 2020;44.
19. Fateh-Moghadam P, Battisti L, Molinaro S, et al. Contact tracing during Phase I of the COVID-19 pandemic in the Province of Trento, Italy: key findings and recommendations. *medRxiv.* 2020.
20. Han T. Outbreak investigation: transmission of COVID-19 starting from a spa facility in a local community in Korea. *Epidemiology and Health.* 2020;0(0):e2020056-2020050.
21. Hu S, Wang W, Wang Y, et al. Infectivity, susceptibility, and risk factors associated with SARS-CoV-2 transmission under intensive contact tracing in Hunan, China. *medRxiv.* 2020.
22. Hua CZ, Miao ZP, Zheng JS, et al. Epidemiological features and viral shedding in children with SARS-CoV-2 infection. *Journal of Medical Virology.* 2020.
23. Islam SS, Noman ASM. Transmission Dynamics and Contact Tracing Assessment of COVID-19 in Chattogram, Bangladesh and Potential Risk of Close Contacts at Different Exposure Settings. *Bangladesh and Potential Risk of Close Contacts at Different Exposure Settings.*
24. Jing Q-L, Liu M-J, Yuan J, et al. Household secondary attack rate of COVID-19 and associated determinants in Guangzhou, China: a retrospective cohort study. *The Lancet Infectious Diseases.* 2020.

© 2020 Madewell ZJ et al. *JAMA Network Open.*

25. Kim J, Choe YJ, Lee J, et al. Role of children in household transmission of COVID-19. *Archives of Disease in Childhood.* 2020:archdischild-2020-319910.
26. Coronavirus Disease-19: Summary of 2,370 Contact Investigations of the First 30 Cases in the Republic of Korea. *Osong Public Health and Research Perspectives.* 2020;11(2):81-84.
27. Laxminarayan R, Wahl B, Dudala SR, et al. Epidemiology and transmission dynamics of COVID-19 in two Indian states. *Science.* 2020.
28. Lee M, Eun Y, Park K, Heo J, Son H. Follow up investigation of asymptomatic COVID-19 cases at diagnosis in Busan, Korea. *Epidemiology and Health.* 2020;0(0):e2020046-2020040.
29. Lewis NM, Chu VT, Ye D, et al. Household transmission of SARS-CoV-2 in the United States. *Clinical Infectious Diseases.* 2020.
30. Li W, Zhang B, Lu J, et al. The characteristics of household transmission of COVID-19. *Clinical Infectious Diseases.* 2020.
31. Liu T, Liang W, Zhong H, et al. Risk factors associated with COVID-19 infection: a retrospective cohort study based on contacts tracing. *Emerging Microbes & Infections.* 2020:1-31.
32. Lopez Bernal J, Panagiotopoulos N, Byers C, et al. Transmission dynamics of COVID-19 in household and community settings in the United Kingdom. *medRxiv.* 2020:2020.2008.2019.20177188.
33. Luo L, Liu D, Liao X, et al. Contact Settings and Risk for Transmission in 3410 Close Contacts of Patients With COVID-19 in Guangzhou, China: A Prospective Cohort Study. *Ann Intern Med.* 2020.
34. Lyngse FP, Kirkeby CT, Halasa T, et al. COVID-19 Transmission Within Danish Households: A Nationwide Study from Lockdown to Reopening. *medRxiv.* 2020.
35. Malheiro R, Figueiredo AL, Magalhães JP, et al. Effectiveness of contact tracing and quarantine on reducing COVID-19 transmission: a retrospective cohort study. *Public Health.* 2020.
36. Park YJ, Choe YJ, Park O, et al. Contact Tracing during Coronavirus Disease Outbreak, South Korea, 2020. *Emerging Infectious Diseases.* 2020;26(10).
37. Park SY, Kim Y-M, Yi S, et al. Coronavirus Disease Outbreak in Call Center, South Korea. *Emerging Infectious Diseases.* 2020;26(8).
38. Patel A, Charani E, Ariyanayagam D, et al. New-onset anosmia and ageusia in adult patients diagnosed with SARS-CoV-2 infection. *Clinical Microbiology and Infection.* 2020.
39. Phiriyasart F, Chantutanon S, Salaeh F, et al. Outbreak Investigation of Coronavirus Disease (COVID-19) among Islamic Missionaries in Southern Thailand, April 2020. *OSIR Journal.* 2020;13(2).
40. Rosenberg ES, Dufort EM, Blog DS, et al. COVID-19 Testing, Epidemic Features, Hospital Outcomes, and Household Prevalence, New York State-March 2020. *Clinical Infectious Diseases.* 2020.
41. Shah K, Desai N, Saxena D, Mavalankar D, Mishra U, Patel GC. Household Secondary Attack Rate in Gandhinagar district of Gujarat state from Western India. *medRxiv.* 2020:2020.2009.2003.20187336.
42. Son H, Lee H, Lee M, et al. Epidemiological characteristics of and containment measures for coronavirus disease 2019 in Busan Metropolitan City, South Korea. *Epidemiology and Health.* 2020:e2020035.
43. Sun WW, Ling F, Pan JR, et al. [Epidemiological characteristics of 2019 novel coronavirus family clustering in Zhejiang Province]. *Zhonghua Yu Fang Yi Xue Za Zhi.* 2020;54(0):E027.
44. Teherani MF, Kao CM, Camacho-Gonzalez A, et al. Burden of illness in households with SARS-CoV-2 infected children. *Journal of the Pediatric Infectious Diseases Society.* 2020.
45. van der Hoek W, Backer JA, Bodewes R, et al. [The role of children in the transmission of SARS-CoV-2]. *Nederlands tijdschrift voor geneeskunde.* 2020;164.
46. Wang Z, Ma W, Zheng X, Wu G, Zhang R. Household transmission of SARS-CoV-2. *The Journal of Infection.* 2020.
47. Wang X, Pan Y, Zhang D, et al. Basic epidemiological parameter values from data of real-world in mega-cities: the characteristics of COVID-19 in Beijing, China. *BMC Infectious Diseases.* 2020;20(1):526.
48. Wang Y, Tian H, Zhang L, et al. Reduction of secondary transmission of SARS-CoV-2 in households by face mask use, disinfection and social distancing: a cohort study in Beijing, China. *BMJ Global Health.* 2020;5(5):e002794.
49. Wang X, Zhou Q, He Y, et al. Nosocomial outbreak of COVID-19 pneumonia in Wuhan, China. *The European Respiratory Journal.* 2020;55(6).
50. Wu J, Huang Y, Tu C, et al. Household Transmission of SARS-CoV-2, Zhuhai, China, 2020. *Clinical Infectious Diseases.* 2020.
51. Wu Y, Song S, Kao Q, Kong Q, Sun Z, Wang B. Risk of SARS-CoV-2 infection among contacts of individuals with COVID-19 in Hangzhou, China. *Public Health.* 2020;185:57-59.

© 2020 Madewell ZJ et al. *JAMA Network Open.*

52. Xin H, Jiang F, Xue A, et al. Risk factors associated with occurrence of COVID-19 among household persons exposed to patients with confirmed COVID-19 in Qingdao Municipal, China. *Transboundary and Emerging Diseases.* 2020.
53. Yu HJ, Hu YF, Liu XX, et al. Household infection: The predominant risk factor for close contacts of patients with COVID-19. *Travel Medicine and Infectious Disease.* 2020;36:101809.
54. Yung CF, Kam KQ, Chong CY, et al. Household Transmission of SARS-CoV-2 from Adults to Children. *The Journal of Pediatrics.* 2020.
55. Zhang W, Cheng W, Luo L, et al. Secondary Transmission of Coronavirus Disease from Presymptomatic Persons, China. *Emerging Infectious Diseases.* 2020;26(8).
56. Zhang JZ, Zhou P, Han DB, et al. [Investigation on a cluster epidemic of COVID-19 in a supermarket in Liaocheng, Shandong province]. *Zhonghua Liu Xing Bing Xue Za Zhi.* 2020;41(0):E055.
57. Zhuang YL, Zhang YT, Li M, et al. [Analysis on the cluster epidemic of coronavirus disease 2019 in Guangdong Province]. *Zhonghua Yu Fang Yi Xue Za Zhi.* 2020;54(7):720-725.
58. Al Hosani FI, Kim L, Khudhair A, et al. Serologic Follow-up of Middle East Respiratory Syndrome Coronavirus Cases and Contacts-Abu Dhabi, United Arab Emirates. *Clinical Infectious Diseases.* 2019;68(3):409-418.
59. Arwady MA, Alraddadi B, Basler C, et al. Middle East respiratory syndrome coronavirus transmission in extended family, Saudi Arabia, 2014. *Emerging Infectious Diseases.* 2016;22(8):1395.
60. Assiri A, McGeer A, Perl TM, et al. Hospital outbreak of Middle East respiratory syndrome coronavirus. *The New England Journal of Medicine.* 2013;369(5):407-416.
61. Drosten C, Meyer B, Müller MA, et al. Transmission of MERS-coronavirus in household contacts. *New England Journal of Medicine.* 2014;371(9):828-835.
62. Memish ZA, Al-Tawfiq JA, Alhakeem RF, et al. Middle East respiratory syndrome coronavirus (MERS-CoV): A cluster analysis with implications for global management of suspected cases. *Travel Medicine and Infectious Disease.* 2015;13(4):311-314.
63. Payne DC, Biggs HM, Al-Abdallat MM, et al. Multihospital Outbreak of a Middle East Respiratory Syndrome Coronavirus Deletion Variant, Jordan: A Molecular, Serologic, and Epidemiologic Investigation. *Open Forum Infectious Diseases.* 2018;5(5):ofy095.
64. Van Kerkhove MD, Alaswad S, Assiri A, et al. Transmissibility of MERS-CoV infection in closed setting, Riyadh, Saudi Arabia, 2015. *Emerging Infectious Diseases.* 2019;25(10):1802.
65. Chan LY, Wong JT, Li PK, Lui SF, Fung H, Sung J. Risk of transmission of severe acute respiratory syndrome to household contacts by infected health care workers and patients. *The American Journal of Medicine.* 2004;116(8):559-560.
66. Goh DL, Lee BW, Chia KS, et al. Secondary household transmission of SARS, Singapore. *Emerg Infect Dis.* 2004;10(2):232-234.
67. Lau JT, Lau M, Kim JH, Tsui HY, Tsang T, Wong TW. Probable secondary infections in households of SARS patients in Hong Kong. *Emerg Infect Dis.* 2004;10(2):235-243.
68. Ou J, Li Q, Zeng G, Dun Z. Efficiency of quarantine during an epidemic of severe acute respiratory syndrome-Beijing, China, 2003. *MMWR: Morbidity & Mortality Weekly Report.* 2003;52(43):1037-1037.
69. Pang X, Zhu Z, Xu F, et al. Evaluation of control measures implemented in the severe acute respiratory syndrome outbreak in Beijing, 2003. *Jama.* 2003;290(24):3215-3221.
70. Tuan PA, Horby P, Dinh PN, et al. SARS transmission in Vietnam outside of the health-care setting. *Epidemiology and Infection.* 2007;135(3):392-401.
71. Wilson-Clark SD, Deeks SL, Gournis E, et al. Household transmission of SARS, 2003. *Canadian Medical Association Journal.* 2006;175(10):1219-1223.
72. Beale S, Lewer D, Aldridge RW, et al. Household transmission of seasonal coronavirus infections: Results from the Flu Watch cohort study. *Wellcome Open Research.* 2020;5(145):145.
73. Esposito S, Bosis S, Niesters HG, et al. Impact of human coronavirus infections in otherwise healthy children who attended an emergency department. *Journal of Medical Virology.* 2006;78(12):1609-1615.
74. Monto AS, DeJonge P, Callear AP, et al. Coronavirus occurrence and transmission over 8 years in the HIVE cohort of households in Michigan. *The Journal of Infectious Diseases.* 2020.
75. Hou H, Wang T, Zhang B, et al. Detection of IgM and IgG antibodies in patients with coronavirus disease 2019. *Clinical & Translational Immunology.* 2020;9(5):e1136.

© 2020 Madewell ZJ et al. *JAMA Network Open.*
